# Supplementary material for: Spatial inequalities in life expectancy within postindustrial regions of Europe: a cross-sectional observational study
Source: BMJ Open. 2014 Jun 2;4(6):e004711. doi: 10.1136/bmjopen-2013-004711 (PMC4054650; doi:10.1136/bmjopen-2013-004711)
Supplement: Author's manuscript [file bmjopen-2013-004711.draft_revisions.pdf]

Spatial inequalities in life expectancy within post-industrial regions of Europe

Journal:	*BMJ Open*
Manuscript ID:	bmjopen-2013-004711
Article Type:	Research
Date Submitted by the Author:	18-Dec-2013

Complete List of Authors:	Taulbut, Martin; NHS Health Scotland,<br>Walsh, David; Glasgow Centre for Population Health,<br>McCartney, Gerry; NHS Greater Glasgow and Clyde<br>Parcell, Sophie; Glasgow Centre for Population Health,<br>Hartmann, Anja; Ruhr-Universität Bochum,<br>Poirier, Gilles; Observatoire Régional de la Santé,<br>Strniskova, Dana; Regional Public Health Authority of the Olomouc Region of the Czech Republic,<br>Hanlon, Phil; University of Glasgow,

**Primary Subject Heading**:	Epidemiology
Secondary Subject Heading:	Public health, Sociology
Keywords:	SOCIAL MEDICINE, PUBLIC HEALTH, STATISTICS & RESEARCH METHODS

SCHOLARONE™
Manuscripts

For peer review only

Spatial inequalities in life expectancy within post-industrial regions of Europe

Martin Taulbut[1], David Walsh[2], Gerry McCartney[3], Sophie Parcell[4], Anja Hartmann[5], Gilles Poirier[6], Dana Strniskova[7], Phil Hanlon[8].

NHS Health Scotland

Meridian Court

5 Cadogan Street

Glasgow G2 6QE.

Scotland.

Tel: +44 0141 414 2754

Correspondence to: martintaulbut@nhs.net

1. NHS Health Scotland, Glasgow, Scotland.
2. Glasgow Centre for Population Health, Glasgow, Scotland.
3. NHS Health Scotland, Glasgow, Scotland.
4. Glasgow Centre for Population Health, Glasgow, Scotland.
5. Ruhr-Universität Bochum Germany.
6. Observatoire Régional de la Santé (ORS), Nord-Pas-de-Calais.
7. Regional Public Health Authority of the Olomouc Region of the Czech Republic.
8. University of Glasgow, Glasgow, Scotland.

2,780 words

For peer review only

Abstract

Objectives To compare spatial inequalities in life expectancy in West Central Scotland (WCS) with nine other post-industrial European regions.

Design A cross-sectional observational study.

Setting West Central Scotland and nine other post-industrial regions across Europe.

Participants Data for WCS and nine other, comparably deindustrialised, European regions were analysed. Male and female life expectancies (LE) at birth were obtained or calculated for the mid-2000s for 160 districts within selected regions. Districts were stratified into two groups: small (populations of between 141,000 and 185,000 people) and large (populations between 224,000 and 352,000). The range and inter-quartile range (IQR) in LE were used to describe within-region disparities.

Results In small districts, the male LE range was widest in WCS and Merseyside, while the IQR was widest in WCS and Northern Ireland. For women, the LE range was widest in WCS, though the IQR was widest in Northern Ireland and Merseyside. In large districts, the range and IQR in life expectancy was widest in WCS and Wallonia for both sexes.

Conclusions Sub-regional spatial inequalities in life expectancy in WCS are wide compared to other post-industrial mainland European regions, especially for men. Future research could explore the contribution of economic, social and political factors in reducing these inequalities.

For peer review only

Strengths and limitations of this study

- This is an extensive, international comparison of contemporary, within-region disparities in life expectancy. It compares 100 small districts and 60 large districts across 10 European regions.
- Ecological bias was mitigated by selecting regions with a similar history of deindustrialisation and comparing districts with similar–sized populations.
- While the approach taken here partly addressed the scale issue associated with the 'Modifiable Area Unit Problem', it was unable to resolve the zoning issue.
- The study was unable to say whether more heterogeneous populations or higher levels of social segregation were driving these differences.
- The analyses are restricted to one period during the mid-to-late 2000s.
- The approach was restricted to describing spatial differences in life expectancy – we cannot draw any conclusions on within-region inequalities by socio-economic status, rurality or ethnicity.

For peer review only

INTRODUCTION

Reducing inequalities in health has been identified as a priority by governments across Europe[1,2]. While inequalities in health are often described using individual characteristics (e.g. socio-economic class), there is also considerable interest in spatial disparities in health[3,4]. All countries exhibit sub-national variation in mortality and life expectancy[5,6,7]. The pattern is observed for countries as diverse as France[8], Sweden[9], Australia[10] and Poland[11]. Almost universally, the geographical gap in these health outcomes is wider for men than women[12]. There are some observed differences in within-country dispersion in life expectancy, with the spatial gap more pronounced for some nations (e.g. USA[13], UK[14]) than others (e.g. Germany[15], Poland[11]). Differences are also observed in whether spatial inequality in mortality has been narrowing, static or increasing over time[16,12], though findings are dependent on the size of geographies selected for analysis[17].

Deindustrialisation has been proposed as a mechanism to partly explain these spatial inequalities. Across Europe, there is a clear overlap between former coalmining and industrial areas and districts and regions with the poorest health[18,6]. A recent study in England found that areas with persistently low or deteriorating employment rates (relative to the national average), often located in ex-industrial regions, had the highest rates of mortality and physical morbidity, even after adjusting for migration and individual characteristics of residents[19]. A number of mechanisms (e.g. greater poverty, loss of purpose and status and higher levels of substance misuse) provide plausible links between economic dislocation and health outcomes[20, 21].

For peer review only

Making spatial comparisons of health within and between geographies is subject to a number of difficulties. Comparing geographies that have been 'clustered' according to some shared characteristics (such as a similar economic and social history) can partly adjust for this and produce more meaningful results[22]. Geographical comparisons are more valid when the spatial units being compared are of a similar population size and where there is less social diversity within them, since the differences between areas will depend on the degree to which the geographical units of analysis are internally diverse or homogeneous. Units of analysis with larger population sizes or more heterogeneity in their composition are less likely to display differences between areas because of the averaging effect of this greater internal diversity[23,17]. Failing to take this into account may result in misleading comparisons.

The present study approaches this issue from a Scottish perspective. Scotland's position as the 'sick man' of Europe – characterised by a slower rate of improvement in life expectancy compared to other West European nations since the 1950s, and a consequent relative deterioration in its international position – has been discussed elsewhere[24,25]. Furthermore, the within-region spatial gap in mortality was greater in Scotland than any other region of Britain[26]. A similar 'faltering' in the pace of improvement in mortality and life expectancy has also been noted for West Central Scotland (WCS), the region of Scotland most affected by deindustrialisation in recent decades, relative to other post-industrial regions[27]. Post-industrial regions are extremely important in epidemiological terms as they tend to exhibit the highest rates of mortality in their parent countries[28,29]. A more recent study also suggested that WCS was more spatially divided in terms of mortality than other, comparable European post-industrial regions, though the authors did not pursues this question in depth[28]. This paper explores this question in a systematic way, to investigate whether spatial disparities in

For peer review only

mortality within WCS are large compared to other European regions, taking both industrial heritage and differences in population sizes of sub-regions into account.

METHODS

This study was informed by the authors' involvement in a larger project which aimed to contribute to an understanding of the poor health observed in one post-industrial region, West Central Scotland, in the context of other comparable European regions. West Central Scotland (WCS) is a region of 2.1 million people, centred on the City of Glasgow. Nine other regions, highlighted in other recent epidemiological analysis[27,29] , were selected for comparison with WCS. The regions were chosen through consultation with experts on European history on the basis of their shared historic economic dependence on industries such as coal, steel, shipbuilding and textiles, alongside analysis of their subsequent loss of industrial employment over the last 30-40 years[27].

Table 1 presents summary information on the list of regions selected. Selecting a range of regions from across East and West Europe allowed contrasts to be made between WCS and European areas with different social and political context. The inclusion of UK regions meant the WCS could be compared with areas subject to the same set of socio-economic policies over the last 30-40 years.

For peer review only

Table 1

Region name	Nation state	Number of districts	Mean population size of districts	Principal historical industries	Total industrial employment loss[a]
West Central Scotland	UK	15[b] (7)[c]	141,268[b] (302,084)[c]	Shipbuilding and support industries (iron, coal, engineering)	-62% (1971-2005)
Northern Ireland	UK	12	147,900	Shipbuilding, textiles, manufacturing	-20% (1971-2005)
Merseyside	UK	9	149,532	Shipping, docks, manufacturing (e.g. cement), engineering	-63% (1971-2005)
Swansea & S. Wales Coalfields	UK	7	160,486	Coal	-51% (1971-2005)
Nord-Pas-de-Calais	UK	25	160,746	Coal, textiles, steel	-43% (1970-2005)
Wallonia	Belgium	11	309,542	Mining, metal working, textiles	-39% (1970-2005)
The Ruhr	Germany	15	351,912	Coal, iron, steel	-54% (1970-2005)
Saxony	Germany	19	224,934	Steel, construction, engineering, textiles	-47% (1991-2005)
Northern Moravia	Czech Republic	11[d]	185,099	Coal, steel	-19% (1993-2005)
Silesia[e]	Poland	29	159,858	Coal, steel, automobiles, zinc	-55% (1980-2005)

a Percentage decrease in the number of industrial jobs in each region over the time period shown in parenthesis. For Silesia, change is shown for the Katowice sub-region.

b Community Health Partnerships.

c Nomenclature of Units for Territorial Statistics (NUTS) 3.

d Jesenik district included in small district comparisons only.

e Known as Slaskie region in Poland.

For peer review only

Male and female life expectancies at birth were obtained from relevant statistical agencies (or where appropriate calculated) for the mid-2000s, for 160 districts within the 10 selected regions. Ideally, the years of the data collected would be of identical time frame and size. It was not possible or practical to do so here, because of variation between countries in terms of availability of the required small area statistics data. All life tables were constructed in the same way, using all deaths within each district and the resident population of each district. The sources of the life expectancy data for each region are given in Table 2 (web-only table).

In order to reduce the risk of bias due to differing sub-regional population sizes (the scale problem), we stratified the regions into two. Five regions (Swansea & S. Wales Coalfields, N. Ireland, Nord-Pas-de-Calais, Silesia and Merseyside) had sub-regional (or district) populations of between 141,000 and 185,000 people. These areas were compared to similarly-sized geographies in WCS Community Health Partnership areas (CHPs).[1] Three regions (the Ruhr, Saxony and Wallonia) had life expectancy data calculated across 45 'large' districts of population size ranging from 224,000 and 352,000: these were compared with similarly-sized WCS Nomenclature of Units for Territorial Statistics (NUTS) 3 areas. Data for Northern Moravia and WCS were available for both strata. For four regions (Northern Ireland, Wallonia, Silesia and Nord-Pas-de-Calais), it was necessary to create pseudo-districts to ensure a more even distribution of population across districts. This process took into account contiguous boundaries and where possible the character of districts. Life expectancy at birth was then calculated for these new areas using the Chiang method (II)[30], using population and mortality data obtained from the relevant national statistical agencies.

[1] There were 15 CHP areas in WCS prior to April 2010, when the five Glasgow CHPs were merged into three.

For peer review only

Within regions, we then ranked the sub-regional (district) populations by their life expectancy separately for men and women and separately for the large and small sub-regional populations. We then created line graphs for each strata of regions to show the size and distribution of sub-regional populations and their corresponding life expectancies. Taking each region separately, we then calculated the range in life expectancy and inter-quartile ranges, accounting for the population sizes in each sub-regional district, to describe the within-regional disparities.

For peer review only

RESULTS

Regions with small district data (populations between 141,000 and 185,000)

The districts with the highest male life expectancies (>77 years at birth) were in the rural districts in Northern Ireland, plus the more affluent WCS districts of East Renfrewshire and East Dunbartonshire. The lowest male life expectancies (<70 years at birth) were in Silesia and in areas of WCS (North and East Glasgow). The districts with the highest levels of female life expectancy (>82.5 years at birth) were all located in Nord-Pas-de Calais whilst the lowest (<78 years at birth) were in WCS (all five Glasgow districts), Merseyside (City & North Liverpool) and parts of the Silesia region (Ruda Slaska & Swietochlowice and Chorzow & Siemianowice Slaskie).

Within regions, the *range* in male life expectancy was widest for WCS (8.6 years) and Merseyside (5.9 years) and narrowest in Swansea & the South Wales Coalfields (1.6 years) and Northern Moravia (2.7 years). The *inter-quartile range* in life expectancy for men was widest in WCS and Northern Ireland (2.7 years and 2.6 years respectively) followed by Silesia (2.2 years) and was much less pronounced in the other regions. For women, WCS had the widest *range* in life expectancy (6.5 years) and Northern Moravia the narrowest (1.6 years). The range of life expectancies observed for Merseyside districts was also high (5.9). The *inter-quartile range* in female life expectancy was highest in Northern Ireland (2.0 years) and Merseyside (1.9 years) and lowest in Northern Moravia (Figure 1).

For peer review only

[Figure 1 about here]

Regions with large district data (populations between 224,000 and 352,000)

The highest male life expectancies were found in Saxony, Wallonia and the Ruhr, whilst the lowest were observed in WCS (Glasgow), Wallonia (Mons) and in Northern Moravia. For women, districts with the highest life expectancy were located in Wallonia and Saxony, while the districts with the lowest life expectancy were found within WCS and Northern Moravia.

Within regions, the *range* in male life expectancy across 'large' districts was widest for WCS (5.3 years) followed by Wallonia (4.8 years), with the Ruhr Valley, Saxony and Northern Moravia less polarised. The *inter-quartile range* in life expectancy was much wider in WCS (3.9 years) than in all other regions. For women, the pattern was similar, with the widest range in life expectancy observed for WCS (3.5 years) and Wallonia (2.5 years), with much less disparity evident in the German and Czech regions (Figure 2).

[Figure 2 about here]

For peer review only

DISCUSSION

Similarly deindustrialised regions in Europe, which share similar economic, social and health problems[27,29], display different patterns in spatial inequalities in life expectancy. In particular, two UK regions (WCS and Merseyside) have much larger intra-regional differences in life expectancy for both men and women than the other regions, with WCS having the largest differences. In contrast, there are relatively narrow spatial inequalities in life expectancy in Northern Moravia, the Ruhr and Swansea and South Wales Coalfields.

The present study has four important strengths. First, it provides an original comparison of contemporary, international, within-region disparities in life expectancy. Second, its geographical coverage is extensive: more than 100 small districts and 60 large districts, spanning 10 regions across both Western and Eastern Europe. Third, it uses a straightforward metric of health outcomes (life expectancy at birth) that is readily understood. Finally, by attempting to ensure the areas are of a similar size and have a common experience of industrial development and subsequent deindustrialisation, the potential bias arising from comparisons of differently sized populations and the heterogeneity within regions is reduced.

The study also has a number of limitations. A key challenge in any study of this kind is the 'Modifiable Area Unit Problem' (MAUP). As discussed by Openshaw (1984)[31], the spatial units that can be used to describe individual level data are usually highly modifiable and their boundaries are often decided on an arbitrary basis. There are a large number of different spatial units that could be used to describe the same data, often producing quite different conclusions. There are two components of the MAUP. First, there is a scale problem, with

For peer review only

different results being produced depending on the number of spatial units used in analysis (e.g. for Census Tracts, districts, regions). Second, there is a grouping or zoning problem, reflecting different choices about how very small areas are joined together to create areas of a similar size. In this study, the scale problem has been partly addressed by making comparisons of sub-regional inequalities at two different geographical levels. The similar findings (of greater spatial inequalities in WCS) for both scales can give more confidence that the approach adopted is reasonable. However, the zoning problem remains difficult to resolve without access to individual-level data coded to geographic areas, which are currently not available.

Data restrictions mean we were unable to explore systematically the degree of social segregation or migration within each region. Spatial inequalities observed could simply reflect greater population heterogeneity between districts within each region. However, evidence comparing WCS with the Ruhr and Nord-Pas-de-Calais does not support this hypothesis[32,33]. Nor can we say how spatial inequalities in LE changed within these regions over time, since the analysis is also confined to a single time period. Lack of individual-level data and common markers of socio-economic status meant this study was also confined to a focus on spatial differences in life expectancy. If data had been available, analysis by inequalities by socio-economic status or other characteristics (e.g. rurality, ethnicity) may have led to different conclusions. For example, in Northern Moravia the gap in male life expectancy between districts was approximately 5 years[34] but the gap in life expectancy between the highest- and least-educated males has been enumerated at16.5 years[35].

For peer review only

The more pronounced spatial inequalities in life expectancy in three of the four UK regions, especially WCS, are notable. What factors might help account for this? As reported elsewhere, despite relatively high levels of mean prosperity and lower unemployment, WCS and the other British regions have higher levels of relative poverty, income inequality and single person and lone parent households compared to post-industrial areas of mainland Europe[29]. There is also a more mixed pattern on some other indicators (e.g. social capital, educational attainment) [29]. It would be appropriate to consider the socio-political context to this. Others have contrasted the UK 'path destructive' road to deindustrialisation, characterised by the growth of a low wage service sector and reduced social protection, with alternative strategies pursued in mainland Europe[21,36]. It has been argued that a more rapid adoption of neoliberal politics by local government in WCS alongside greater vulnerability to the deleterious impacts of associated economic policies might provide some basis for explaining the findings for WCS[21, 37]

It may be that in other countries, 'protective' factors such as lower levels of income inequality (Northern Moravia)[38], higher levels of social capital (The Ruhr)[32] or fewer lone parent or single person households (Nord Pas de Calais)[33], or a more managed deindustrialisation process which included active labour market policies and re-employment in new industrial sectors[21], might have partly mitigated against the health-damaging effects of deindustrialisation, reducing the extent of spatial inequalities in health. However, as yet unexplained region-specific factors are also likely to play a role. Within the UK, Swansea and South Wales has relatively narrow spatial inequalities in health and WCS has some of the widest. In the former case, this may partly reflect the more homogenous social mix across ex-mining areas/villages, compared to more metropolitan areas.

For peer review only

Difference in lifestyle factors (i.e. worse health behaviours in WCS) could also play a role. This argument is more plausible for alcohol, since levels of consumption and alcohol-related harm are high in WCS for both genders compared to the other regions[29]. For smoking and diet, matters are less clear. Female smoking rates are higher in West Central Scotland compared to most regions but male smoking rates are similar across all regions[29]. Dietary indicators suggest WCS compares poorly with Nord-Pas-de-Calais but is very similar to Merseyside and Northern Ireland[28]. That said, any explanation based on health behaviours alone would be insufficient, as the underlying causes of these health behaviours would remain unexplained.

CONCLUSIONS

Sub-regional spatial inequalities in life expectancy in West Central Scotland are wide compared to other post-industrial European regions, even after accounting for differences in the population size of the sub-regional districts. These spatial inequalities are particularly profound for men. By contrast, within-region spatial inequalities in life expectancy were relatively low in the German and Czech regions. These data generally show similar patterns to that for inequalities by individual educational attainment for the parent countries[39]. Outside the UK, wider determinants of health (such as income distribution, positive social capital and family networks) may have acted to protect health in post-industrial regions. Future research could explore the contribution of these wider determinants of health to reducing spatial inequalities in mortality, especially in West Central Scotland.

For peer review only

ACKNOWLEDGEMENTS

This project would not have been possible without the cooperation and assistance of a number of individuals and organisations. In particular, we would like to thank the following for all their help in providing the required data:

- National Records for Scotland (NRS), formerly General Register Office for Scotland.
- Northern Ireland Statistics and Research Agency.
- Office for National Statistics and Public Health Intelligence Team, Information & Intelligence Services, Liverpool Primary Care Trust.
- Office for National Statistics (Vital Statistics).
- INSEE and Centre d'épidémiologie sur les causes médicales de décès (CepiDc) (Orignal data provided by Observatoire Régional de la Santé (ORS), NPdC).
- SPMA (https://www.wiv-isp.be/epidemio/spma), Public Health and Surveillance, Scientific Institute of Public Health, Brussels, Belgium.
- NRW Institute of Health and Work (LIGA.NRW).
- Statistisches Landesamt des Freistaates Sachsen.
- Czech Statistical Office.
- Central Statistical Office of Poland – Local Data Bank.

All underlying data used remains © to the relevant agencies.

Please note that the mortality and population data for Saxony are © Statistisches Landesamt des Freistaates Sachsen, Kamenz, 2007.

CONFLICTS OF INTEREST

None declared. Gerry McCartney is a member of the Scottish Socialist Party.

FUNDING

GM and DW conceived the idea for the paper and designed the study. All authors were involved in the acquisition of data, its analysis and interpretation. All authors contributed to the drafting and revising of the paper.

For peer review only

REFERENCES

1. Scottish Government. Equally Well: Report of the Ministerial Task Force on Health Inequalities. Edinburgh: The Scottish Government; 2008.

2. Mackenbach JP. Health Inequalities: Europe in Profile. Rotterdam: Erasmus MC; 2006.

3. Shaw M, Thomas B, Davey Smith G, Dorling D. The Grim Reaper's road map: An atlas of mortality in Britain. Bristol: The Policy Press; 2008.

4. Bonneux LG, Huisman CC, de Beer JA. Mortality in 272 European regions, 2002-2004. An update. European Journal of Epidemiology 2010;25(2):77-85.

5. Ezzati M, Friedman AB, Kulkarni SC, Murray CJL. The reversal of fortunes: Trends in county mortality and cross-county mortality disparities in the United States. PLoS Med 2008;5(4):557-568.

6. European Commission. Health statistics – Atlas on mortality in the European Union. Luxembourg: Office for Official Publications of the European Communities; 2009.

7. Suzuki E, Kashima S, Kawachi I, Subramanian SV. Social and geographic inequalities in premature adult mortality in Japan: a multilevel observational study from 1970 to 2005. BMJ Open 2012;2:e000425 doi:10 1136/bmjopen-2011-000425 .

8. Windenberger F, Rican S, Jougla E, Rey G. Spatiotemporal association between deprivation and mortality: trends in France during the nineties Eur J Public Health 2012;22(3):347-353.

9. Statistics Sweden. Demographic reports 2007:1, Life expectancy in Sweden 2001–2005. Life expectancy tables for the country and by county. Stockholm: Statistics Sweden; 2007.

For peer review only

10. Australian Bureau of Statistics. Feature Article - Regional mortality. 2002; Available at: http://www.abs.gov.au/ausstats/abs@.nsf/featurearticlesbytitle/5D0B63A4CED5187DCA256C8C007DF565?OpenDocument. Accessed August 23, 2012.

11. GUS. Life Expectancy Tables of Poland 2010. Warsaw: GUS; 2011.

12. Vallin J, Mesle F, Valkonen T. Trends in mortality and differential mortality. Strasbourg: Council of Europe; 2001.

13. Murray CJL, Michaud CM, McKenna MT, Marks JS. U.S. Patterns of Mortality by County and Race: 1965-94. Cambridge MA: Harvard Center for Population and Development Studies; 1998.

14. Office for National Statistics. Life expectancy at birth and at age 65 by local areas in the United Kingdom, 2004–06 to 2008–10. Newport: ONS.

15. Edenharter G, Haas K, Schober-Halstenberg HJ, Frei U. Regional Life Expectancy in Germany: Life Table Analysis for 1997/1999 to 2004/2006. Part A: Methods and Summary of Results. 2009; Available at: http://www.edenharter-research.de/regle_de/pdf/RegLE_DE_PartA.pdf. Accessed August 23, 2012.

16. Dorling D. Death in Britain: how local mortality rates have changed: 1950s - 1990s. York: Joseph Rowntree Foundation.; 1997.

17. Jackson AL, Davies CA, Leyland AH. Do differences in the administrative structure of populations confound comparisons of geographic health inequalities? BMC Medical Research Methodology 2010;10:74.

18. Riva M, Terashima M, Curtis S, Shucksmith J, Carlebach S. Coalfield health effects: variation in health across former coal field areas in England. Health Place 2011;17:588e97.

For peer review only

19. Riva M, Curtis SE. Long-term local area employment rates as predictors of individual mortality and morbidity: a prospective study in England, spanning more than two decades. J Epidemiol Community Health 2012, 66, 919-926.

20. Bartley M. Unemployment and ill health: understanding the relationship. J Epidemiol Community Health 1994; 48:333e7.

21. Collins C, McCartney G. The impact of neoliberal 'political attack' on health: the case of the 'Scottish Effect'. Int J Health Services 2011;41(3):501-513.

22. Application of cluster analysis towards the development of health region peer groups. Statistical Society of Canada, Annual Meeting; June 2003; 2003.

23. Shaw M, Orford S, Brimblecombe N, Dorling D. Widening inequality in mortality between 160 regions of 15 European countries in the early 1990s. Social Science & Medicine ;50:1047-1058.

24. Whyte B. Scottish mortality in a European context 1950 - 2000 An analysis of comparative mortality trends. Edinburgh: Information Services, NSS; 2006.

25. McCartney G, Walsh D, Whyte B, Collins C. Has Scotland always been the 'sick man' of Europe? An observational study from 1855 to 2006. Eur J Public Health 2011.

26. Leyland A. Increasing inequalities in premature mortality in Great Britain. J Epidemiol Community Health 2004;58:296-302.

27. Walsh D, Taulbut M, Hanlon P. The aftershock of deindustrialization—trends in mortality in Scotland and other parts of post-industrial Europe. Eur J Public Health 2010;20:58-64.

For peer review only

28. Taulbut M, Walsh D, Parcell S, et al. Health and its determinants in Scotland and other parts of post-industrial Europe: The Aftershock of Deindustrialisation Study phase two. Glasgow: GCPH; 2011.

29. Taulbut M, Walsh D, Parcell S, et al. What can ecological data tell us about reasons for divergence in health status between West Central Scotland and other regions of post-industrial Europe?, Public Health; 2012http://dx.doi.org/10.1016/j.puhe.2012.11.007

30. Chiang CL. The Life Table and Mortality Analysis. Geneva: World Health Organization; 1978.

31. Openshaw S. The Modifiable Areal Unit Problem. Norwich: Geo Books; 1984.

32. Parcell S, Hartmann A, Taulbut M, Walsh D. Case study: Health and its determinants in West Central Scotland compared to the Ruhr area in Germany. Glasgow: GCPH; 2011.

33. Taulbut M, Poirier G, Walsh D. Case study: Health and its determinants in West Central Scotland compared to Nord-Pas-de-Calais in France. Glasgow: GCPH; 2011.

34. CZSO. Life expectancy in districts (LAU 1) in 2005-2009 (data extra regular outputs). Available at: http://www.czso.cz/eng/redakce.nsf/i/life_tables. Accessed August

35. van Raalte AA, Kunst AE, Lundberg O, Leinsalu M, Martikainen P, Artnik B, et al. The contribution of educational inequalities to lifespan variation. Population Health Metrics 2012;10(3).

36. Birch K, Mykhnenko K. Varieties of neoliberalism? Restructuring in large industrially dependent regions across Western and Eastern Europe. Journal of Economic Geography 2009; 9: 355–380.

37. Boyle M, McWilliams C, Rice G. The spatialities of actually existing neoliberalism in Glasgow, 1977 to present. 2008;90(4):313-325.

For peer review only

38. Walsh D, Strniskova D, Taulbut M. Case study: Health and its determinants in West Central Scotland compared to Northern Moravia in the Czech Republic. Glasgow: GCPH; 2011.

39. Popham F, Boyle P. Assessing socio-economic inequalities in mortality and other health outcomes at the Scottish national level. Edinburgh: Scottish Collaboration for Public Health Research and Policy; 2011.

For peer review only

Figure 1 – Sub-regional disparities in female and male life expectancy within European industrialised regions (including regions with sub-regional data for populations of 185,000 people or less)

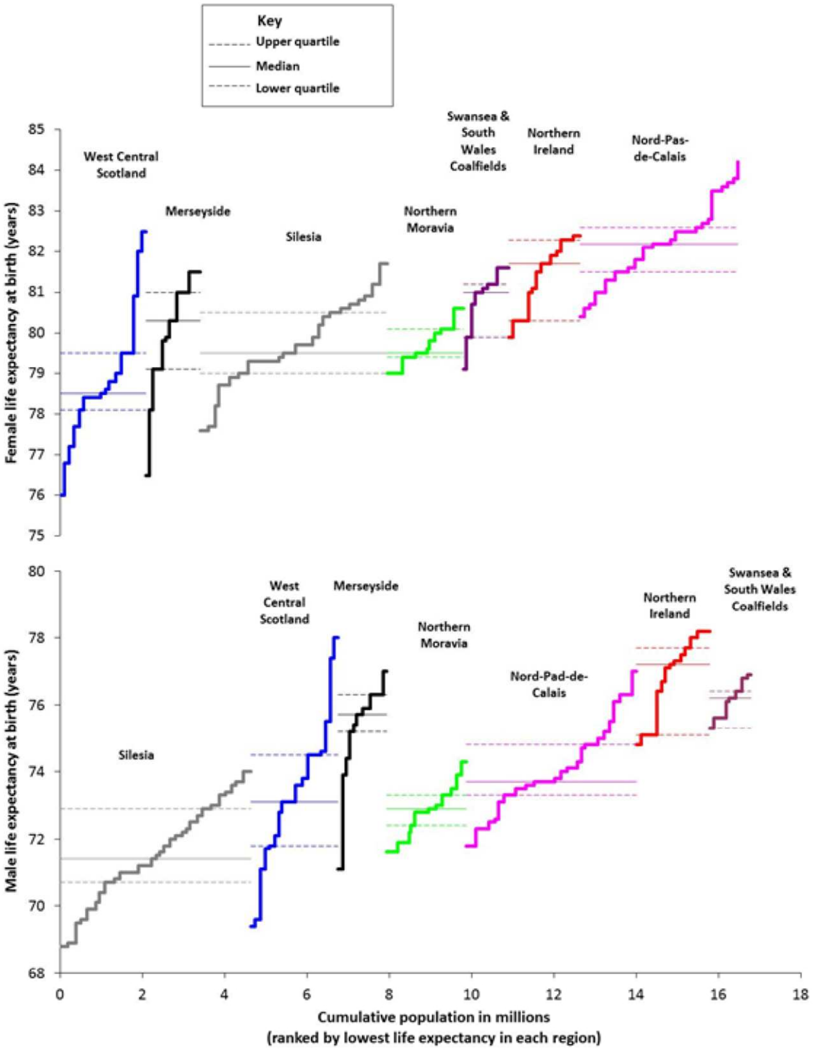

157x217mm (200 x 200 DPI)

Figure 2 – Sub-regional disparities in female and male life expectancy within European industrialised regions (including regions with sub-regional data for populations of 224,000 people or more)

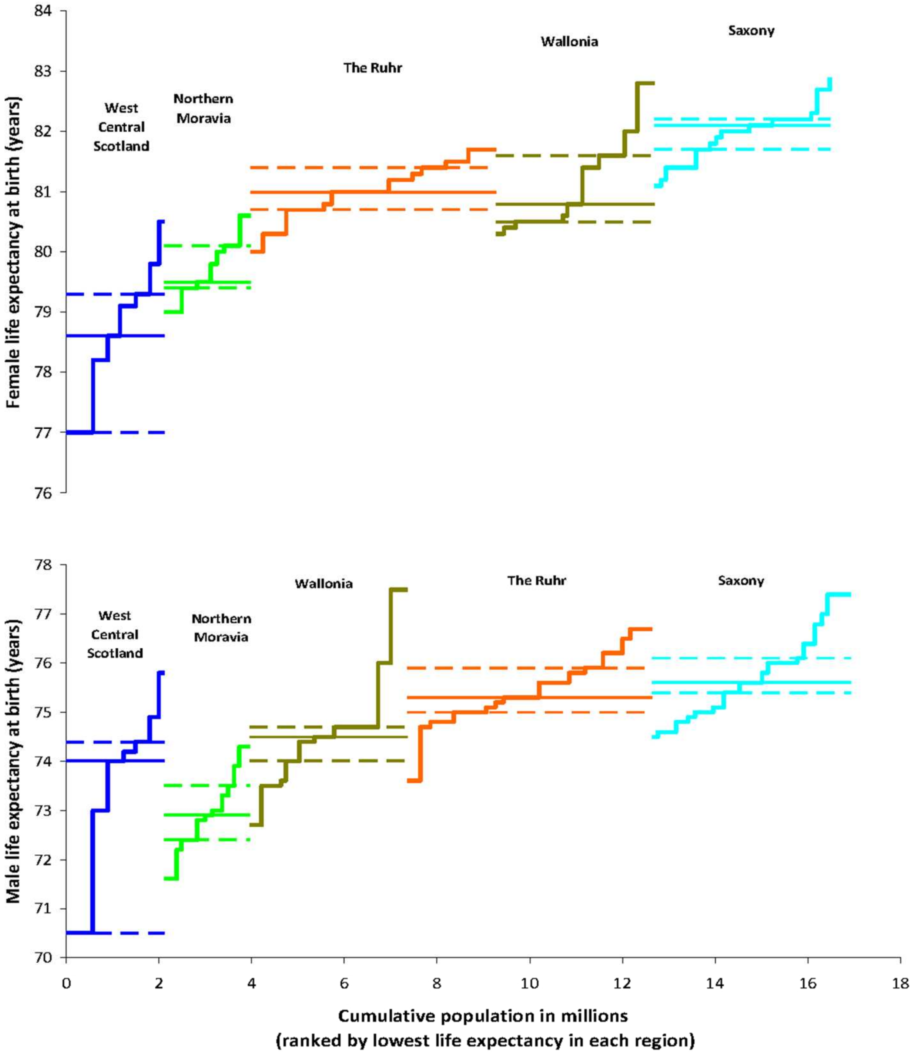

152x192mm (200 x 200 DPI)

STROBE Statement—checklist of items that should be included in reports of observational studies

	Item No	Recommendation
**Title and abstract**	1	(*a*) Indicate the study's design with a commonly used term in the title or the abstract
		(*b*) Provide in the abstract an informative and balanced summary of what was done and what was found
**Introduction**		
Background/rationale	2	Explain the scientific background and rationale for the investigation being reported
Objectives	3	State specific objectives, including any prespecified hypotheses
**Methods**		
Study design	4	Present key elements of study design early in the paper
Setting	5	Describe the setting, locations, and relevant dates, including periods of recruitment, exposure, follow-up, and data collection
Participants	6	(*a*) *Cohort study*—Give the eligibility criteria, and the sources and methods of selection of participants. Describe methods of follow-up<br>*Case-control study*—Give the eligibility criteria, and the sources and methods of case ascertainment and control selection. Give the rationale for the choice of cases and controls<br>*Cross-sectional study*—Give the eligibility criteria, and the sources and methods of selection of participants
		(*b*) *Cohort study*—For matched studies, give matching criteria and number of exposed and unexposed<br>*Case-control study*—For matched studies, give matching criteria and the number of controls per case
Variables	7	Clearly define all outcomes, exposures, predictors, potential confounders, and effect modifiers. Give diagnostic criteria, if applicable
Data sources/ measurement	8*	For each variable of interest, give sources of data and details of methods of assessment (measurement). Describe comparability of assessment methods if there is more than one group
Bias	9	Describe any efforts to address potential sources of bias
Study size	10	Explain how the study size was arrived at
Quantitative variables	11	Explain how quantitative variables were handled in the analyses. If applicable, describe which groupings were chosen and why
Statistical methods	12	(*a*) Describe all statistical methods, including those used to control for confounding
		(*b*) Describe any methods used to examine subgroups and interactions
		(*c*) Explain how missing data were addressed
		(*d*) *Cohort study*—If applicable, explain how loss to follow-up was addressed<br>*Case-control study*—If applicable, explain how matching of cases and controls was addressed<br>*Cross-sectional study*—If applicable, describe analytical methods taking account of sampling strategy
		(*e*) Describe any sensitivity analyses

Continued on next page

For peer review only

**Results**		
Participants	13*	(a) Report numbers of individuals at each stage of study—eg numbers potentially eligible, examined for eligibility, confirmed eligible, included in the study, completing follow-up, and analysed
		(b) Give reasons for non-participation at each stage
		(c) Consider use of a flow diagram
Descriptive data	14*	(a) Give characteristics of study participants (eg demographic, clinical, social) and information on exposures and potential confounders
		(b) Indicate number of participants with missing data for each variable of interest
		(c) *Cohort study*—Summarise follow-up time (eg, average and total amount)
Outcome data	15*	*Cohort study*—Report numbers of outcome events or summary measures over time
		Case-control study—Report numbers in each exposure category, or summary measures of exposure
		Cross-sectional study—Report numbers of outcome events or summary measures
Main results	16	(*a*) Give unadjusted estimates and, if applicable, confounder-adjusted estimates and their precision (eg, 95% confidence interval). Make clear which confounders were adjusted for and why they were included
		(*b*) Report category boundaries when continuous variables were categorized
		(*c*) If relevant, consider translating estimates of relative risk into absolute risk for a meaningful time period
Other analyses	17	Report other analyses done—eg analyses of subgroups and interactions, and sensitivity analyses
**Discussion**		
Key results	18	Summarise key results with reference to study objectives
Limitations	19	Discuss limitations of the study, taking into account sources of potential bias or imprecision. Discuss both direction and magnitude of any potential bias
Interpretation	20	Give a cautious overall interpretation of results considering objectives, limitations, multiplicity of analyses, results from similar studies, and other relevant evidence
Generalisability	21	Discuss the generalisability (external validity) of the study results
**Other information**		
Funding	22	Give the source of funding and the role of the funders for the present study and, if applicable, for the original study on which the present article is based

*Give information separately for cases and controls in case-control studies and, if applicable, for exposed and unexposed groups in cohort and cross-sectional studies.

Note: An Explanation and Elaboration article discusses each checklist item and gives methodological background and published examples of transparent reporting. The STROBE checklist is best used in conjunction with this article (freely available on the Web sites of PLoS Medicine at http://www.plosmedicine.org/, Annals of Internal Medicine at http://www.annals.org/, and Epidemiology at http://www.epidem.com/). Information on the STROBE Initiative is available at www.strobe-statement.org.

For peer review only

For peer review only - http://bmjopen.bmj.com/site/about/guidelines.xhtml

Spatial inequalities in life expectancy within post-industrial regions of Europe: a cross-sectional observational study

Journal:	*BMJ Open*
Manuscript ID:	bmjopen-2013-004711.R1
Article Type:	Research
Date Submitted by the Author:	06-May-2014
Complete List of Authors:	Taulbut, Martin; NHS Health Scotland,<br>Walsh, David; Glasgow Centre for Population Health,<br>McCartney, Gerry; NHS Greater Glasgow and Clyde<br>Parcell, Sophie; Glasgow Centre for Population Health,<br>Hartmann, Anja; Ruhr-Universität Bochum,<br>Poirier, Gilles; Observatoire Régional de la Santé,<br>Strniskova, Dana; Regional Public Health Authority of the Olomouc Region of the Czech Republic,<br>Hanlon, Phil; University of Glasgow,
**Primary Subject Heading**:	Epidemiology
Secondary Subject Heading:	Public health, Sociology
Keywords:	SOCIAL MEDICINE, PUBLIC HEALTH, STATISTICS & RESEARCH METHODS

SCHOLARONE™
Manuscripts

Spatial inequalities in life expectancy within post-industrial regions of Europe: a cross-sectional observational study

Martin Taulbut[1], David Walsh[2], Gerry McCartney[3], Sophie Parcell[4], Anja Hartmann[5], Gilles Poirier[6], Dana Strniskova[7], Phil Hanlon[8].

NHS Health Scotland

Meridian Court

5 Cadogan Street

Glasgow G2 6QE.

Scotland.

Tel: +44 0141 414 2754

Correspondence to: martintaulbut@nhs.net

1. NHS Health Scotland, Glasgow, Scotland.
2. Glasgow Centre for Population Health, Glasgow, Scotland.
3. NHS Health Scotland, Glasgow, Scotland.
4. Glasgow Centre for Population Health, Glasgow, Scotland.
5. Ruhr-Universität Bochum Germany.
6. Observatoire Régional de la Santé (ORS), Nord-Pas-de-Calais.
7. Regional Public Health Authority of the Olomouc Region of the Czech Republic.
8. University of Glasgow, Glasgow, Scotland.

3,500 words

For peer review only

Abstract

Objectives To compare spatial inequalities in life expectancy in West Central Scotland (WCS) with nine other post-industrial European regions.

Design A cross-sectional observational study.

Setting West Central Scotland and nine other post-industrial regions across Europe.

Participants Data for WCS and nine other, comparably deindustrialised, European regions were analysed. Male and female life expectancies (LE) at birth were obtained or calculated for the mid-2000s for 160 districts within selected regions. Districts were stratified into two groups: small (populations of between 141,000 and 185,000 people) and large (populations between 224,000 and 352,000). The range and inter-quartile range (IQR) in LE were used to describe within-region disparities.

Results In small districts, the male LE range was widest in WCS and Merseyside, while the IQR was widest in WCS and Northern Ireland. For women, the LE range was widest in WCS, though the IQR was widest in Northern Ireland and Merseyside. In large districts, the range and IQR in life expectancy was widest in WCS and Wallonia for both sexes.

Conclusions Sub-regional spatial inequalities in life expectancy in WCS are wide compared to other post-industrial mainland European regions, especially for men. Future research could explore the contribution of economic, social and political factors in reducing these inequalities.

For peer review only

Strengths and limitations of this study

- This is an extensive, international comparison of contemporary, within-region disparities in life expectancy. It compares 100 small districts and 60 large districts across 10 European regions.
- Ecological bias was mitigated by selecting regions with a similar history of deindustrialisation and comparing districts with similar–sized populations.
- While the approach taken here partly addressed the scale issue associated with the 'Modifiable Area Unit Problem', it was unable to resolve the zoning issue.
- The study was unable to say whether more heterogeneous populations or higher levels of social segregation were driving these differences, though the limited evidence we have does not support this view.
- The analyses are restricted to one period during the mid-to-late 2000s.
- The approach was restricted to describing spatial differences in life expectancy – we cannot draw any conclusions on within-region inequalities by socio-economic status, rurality or ethnicity.

For peer review only

INTRODUCTION

Reducing inequalities in health has been identified as a priority by governments across Europe[1,2]. While inequalities in health are often described using individual characteristics (e.g. socio-economic class), there is also considerable interest in spatial disparities in health[3,4], despite a lack of research found by Tyner et al. (2014)[5]. All countries exhibit sub-national variation in mortality and life expectancy[6,7,8]. The pattern is observed for countries as diverse as France[9], Sweden[10], Australia[11] and Poland[12]. Almost universally, the geographical gap in these health outcomes is wider for men than women[13]. There are some observed differences in within-country dispersion in life expectancy, with the spatial gap more pronounced for some nations (e.g. USA[14], UK[15]) than others (e.g. Germany[16], Poland[12]). Regional inequalities in mortality between English regions, for instance, have been found to be severe and persistent over a forty-year period[17]. Differences are also observed in whether spatial inequality in mortality has been narrowing, static or increasing over time[18,13]. Although findings are dependent on the size of geographies selected for analysis[19,], there is evidence that inequalities both between and within English regions have increased over time[17,20].

Deindustrialisation has been proposed as a mechanism to partly explain these spatial inequalities. Across Europe, there is a clear overlap between former coalmining and industrial areas and districts and regions with the poorest health[21,7]. Riva et al. (2012) found that areas in England with persistently low or deteriorating employment rates (relative to the national average), often located in ex-industrial regions, had the highest rates of mortality and physical morbidity, even after adjusting for migration and individual characteristics of residents[22]. A number of mechanisms (e.g. greater poverty, loss of purpose and status and

For peer review only

higher levels of substance misuse) provide plausible links between economic dislocation and health outcomes[23, 24].

Making spatial comparisons of health within and between geographies is subject to a number of difficulties. Comparing geographies that have been 'clustered' according to some shared characteristics (such as a similar economic and social history) can partly adjust for this and produce more meaningful results[25]. Geographical comparisons are more valid when the spatial units being compared are of a similar population size and where there is less social diversity within them, since the differences between areas will depend on the degree to which the geographical units of analysis are internally diverse or homogeneous. Units of analysis with larger population sizes or more heterogeneity in their composition are less likely to display differences between areas because of the averaging effect of this greater internal diversity[26,19]. Failing to take this into account may result in misleading comparisons.

The present study approaches this issue from a Scottish perspective. Scotland's position as the 'sick man' of Europe – characterised by a slower rate of improvement in life expectancy compared to other West European nations since the 1950s, and a consequent relative deterioration in its international position – has been discussed elsewhere[27,28]. Furthermore, the within-region spatial gap in mortality was greater in Scotland than any other region of Britain[29]. A similar 'faltering' in the pace of improvement in mortality and life expectancy has also been noted for West Central Scotland (WCS), the region of Scotland most affected by deindustrialisation in recent decades, relative to other post-industrial regions[30]. Post-industrial regions are extremely important in epidemiological terms as they tend to exhibit the highest rates of mortality in their parent countries[31,32]. A more recent study also suggested

For peer review only

that WCS was more spatially divided in terms of mortality than other, comparable European post-industrial regions, though the authors did not pursues this question in depth[31]. This paper explores this question in a systematic way, to investigate whether spatial disparities in mortality within WCS are large compared to other European regions, taking both industrial heritage and differences in population sizes of sub-regions into account.

METHODS

This study was informed by the authors' involvement in a larger project which aimed to contribute to an understanding of the poor health observed in one post-industrial region, West Central Scotland, in the context of other comparable European regions. West Central Scotland (WCS) is a region of 2.1 million people, centred on the City of Glasgow. Nine other regions, highlighted in other recent epidemiological analysis[30,32] , were selected for comparison with WCS. The regions were chosen through consultation with experts on European history on the basis of their shared historic economic dependence on industries such as coal, steel, shipbuilding and textiles, alongside analysis of their subsequent loss of industrial employment over the last 30-40 years[30].

Table 1 presents summary information on the list of regions selected. Selecting a range of regions from across East and West Europe allowed contrasts to be made between WCS and European areas with different social and political context. The inclusion of UK regions meant the WCS could be compared with areas subject to the same set of socio-economic policies over the last 30-40 years.

For peer review only

Table 1

Region name	Nation state	Number of districts	Mean population size of districts	Principal historical industries	Total industrial employment loss[a]
West Central Scotland	UK	15[b] (7)[c]	141,268[b] (302,084)[c]	Shipbuilding and support industries (iron, coal, engineering)	-62% (1971-2005)
Northern Ireland	UK	12	147,900	Shipbuilding, textiles, manufacturing	-20% (1971-2005)
Merseyside	UK	9	149,532	Shipping, docks, manufacturing (e.g. cement), engineering	-63% (1971-2005)
Swansea & S. Wales Coalfields	UK	7	160,486	Coal	-51% (1971-2005)
Nord-Pas-de-Calais	France	25	160,746	Coal, textiles, steel	-43% (1970-2005)
Wallonia	Belgium	11	309,542	Mining, metal working, textiles	-39% (1970-2005)
The Ruhr	Germany	15	351,912	Coal, iron, steel	-54% (1970-2005)
Saxony	Germany	19	224,934	Steel, construction, engineering, textiles	-47% (1991-2005)
Northern Moravia	Czech Republic	11[d]	185,099	Coal, steel	-19% (1993-2005)
Silesia[e]	Poland	29	159,858	Coal, steel, automobiles, zinc	-55% (1980-2005)

a Percentage decrease in the number of industrial jobs in each region over the time period shown in parenthesis. For Silesia, change is shown for the Katowice sub-region.

b Community Health Partnerships.

c Nomenclature of Units for Territorial Statistics (NUTS) 3.

d Jesenik district included in small district comparisons only.

e Known as Slaskie region in Poland.

For peer review only

Male and female life expectancies at birth were obtained from relevant statistical agencies (or where appropriate calculated) for the mid-2000s, for 160 districts within the 10 selected regions. Ideally, the years of the data collected would be of identical time frame and size. It was not possible or practical to do so here, because of variation between countries in terms of availability of the required small area statistics data. All life tables were constructed in the same way, using all deaths within each district and the resident population of each district. The sources of the life expectancy data for each region are given in Table 2 (web-only table).

In order to reduce the risk of bias due to differing sub-regional population sizes (the scale problem), we stratified the regions into two. Five regions (Swansea & S. Wales Coalfields, N. Ireland, Nord-Pas-de-Calais, Silesia and Merseyside) had sub-regional (or district) populations of between 141,000 and 185,000 people. These areas were compared to similarly-sized geographies in WCS Community Health Partnership areas (CHPs).[1] Three regions (the Ruhr, Saxony and Wallonia) had life expectancy data calculated across 45 'large' districts of population size ranging from 224,000 and 352,000: these were compared with similarly-sized WCS Nomenclature of Units for Territorial Statistics (NUTS) 3 areas. Data for Northern Moravia and WCS were available for both strata. For four regions (Northern Ireland, Wallonia, Silesia and Nord-Pas-de-Calais), it was necessary to create pseudo-districts to ensure a more even distribution of population across districts. This process took into account contiguous boundaries and where possible the character of districts. Life expectancy at birth was then calculated for these new areas using the Chiang method (II)[33], using population and mortality data obtained from the relevant national statistical agencies.

[1] There were 15 CHP areas in WCS prior to April 2010, when the five Glasgow CHPs were merged into three.

For peer review only

Within regions, we then ranked the sub-regional (district) populations by their life expectancy separately for men and women and separately for the large and small sub-regional populations. We then created line graphs for each strata of regions to show the size and distribution of sub-regional populations and their corresponding life expectancies. Taking each region separately, we then calculated the range in life expectancy and inter-quartile ranges, accounting for the population sizes in each sub-regional district, to describe the within-regional disparities.

For peer review only

RESULTS

Regions with small district data (populations between 141,000 and 185,000)

The districts with the highest male life expectancies (>77 years at birth) were in the rural districts in Northern Ireland, plus the more affluent WCS districts of East Renfrewshire and East Dunbartonshire. The lowest male life expectancies (<70 years at birth) were in Silesia and in areas of WCS (North and East Glasgow). The districts with the highest levels of female life expectancy (>82.5 years at birth) were all located in Nord-Pas-de Calais whilst the lowest (<78 years at birth) were in WCS (all five Glasgow districts), Merseyside (City & North Liverpool) and parts of the Silesia region (Ruda Slaska & Swietochlowice and Chorzow & Siemianowice Slaskie).

Within regions, the *range* in male life expectancy was widest for WCS (8.6 years) and Merseyside (5.9 years) and narrowest in Swansea & the South Wales Coalfields (1.6 years) and Northern Moravia (2.7 years). The *inter-quartile range* in life expectancy for men was widest in WCS and Northern Ireland (2.7 years and 2.6 years respectively) followed by Silesia (2.2 years) and was much less pronounced in the other regions. For women, WCS had the widest *range* in life expectancy (6.5 years) and Northern Moravia the narrowest (1.6 years). The range of life expectancies observed for Merseyside districts was also high (5.9). The *inter-quartile range* in female life expectancy was highest in Northern Ireland (2.0 years) and Merseyside (1.9 years) and lowest in Northern Moravia (Figure 1).

For peer review only - http://bmjopen.bmj.com/site/about/guidelines.xhtml

[Figure 1 about here]

Regions with large district data (populations between 224,000 and 352,000)

The highest male life expectancies were found in Saxony, Wallonia and the Ruhr, whilst the lowest were observed in WCS (Glasgow), Wallonia (Mons) and in Northern Moravia. For women, districts with the highest life expectancy were located in Wallonia and Saxony, while the districts with the lowest life expectancy were found within WCS and Northern Moravia.

Within regions, the *range* in male life expectancy across 'large' districts was widest for WCS (5.3 years) followed by Wallonia (4.8 years), with the Ruhr Valley, Saxony and Northern Moravia less polarised. The *inter-quartile range* in life expectancy was much wider in WCS (3.9 years) than in all other regions. For women, the pattern was similar, with the widest range in life expectancy observed for WCS (3.5 years) and Wallonia (2.5 years), with much less disparity evident in the German and Czech regions (Figure 2).

[Figure 2 about here]

For peer review only

DISCUSSION

Similarly deindustrialised regions in Europe, which share similar economic, social and health problems[30,32], display different patterns in spatial inequalities in life expectancy. In particular, two UK regions (WCS and Merseyside) have much larger intra-regional differences in life expectancy for both men and women than the other regions, with WCS having the largest differences. In contrast, there are relatively narrow spatial inequalities in life expectancy in Northern Moravia, the Ruhr and Swansea and South Wales Coalfields.

The present study has four important strengths. First, it provides an original comparison of contemporary, international, within-region disparities in life expectancy. Second, its geographical coverage is extensive: more than 100 small districts and 60 large districts, spanning 10 regions across both Western and Eastern Europe. Third, it uses a straightforward metric of health outcomes (life expectancy at birth) that is readily understood. Finally, by attempting to ensure the areas are of a similar size and have a common experience of industrial development and subsequent deindustrialisation, the potential bias arising from comparisons of differently sized populations and the heterogeneity within regions is reduced.

The study also has a number of limitations. A key challenge in any study of this kind is the 'Modifiable Area Unit Problem' (MAUP). As discussed by Openshaw (1984)[34], the spatial units that can be used to describe individual level data are usually highly modifiable and their boundaries are often decided on an arbitrary basis. There are a large number of different spatial units that could be used to describe the same data, often producing quite different conclusions. There are two components of the MAUP. First, there is a scale problem, with

For peer review only

different results being produced depending on the number of spatial units used in analysis (e.g. for Census Tracts, districts, regions). Second, there is a grouping or zoning problem, reflecting different choices about how very small areas are joined together to create areas of a similar size. In this study, the scale problem has been partly addressed by making comparisons of sub-regional inequalities at two different geographical levels. The similar findings (of greater spatial inequalities in WCS) for both scales can give more confidence that the approach adopted is reasonable. However, the zoning problem remains difficult to resolve without access to individual-level data coded to geographic areas, which are currently not available. It is important to note that the findings may not apply beyond the selection of post-industrial regions shown here. For example, Hoffman et (2012), who analysed neighbourhood-level differences in mortality for 15 large European large cities, found that inequalities were wider for women than for men, and no evidence that within-area inequalities varied between cities[35].

The methods used to compare spatial inequalities (interquartile range) could also be criticised as not ideal. Other studies[36] have used the slope index of inequality (SII) and relative index of inequality (RII) to estimate spatial inequalities in mortality[37]. This would undoubtedly allow for more robust analyses. However, to allow these indices to be constructed would require robust, internationally comparable measures for ranking all the districts by socio-economic status. Data limitations make this a difficult task. Europe-wide indicators of material and income deprivation are unavailable for small-area geographies. A prototype European Socio-economic Classification[38] has been developed but comparable small area data (from national Censuses) for all areas are not yet available. Limited measures of housing tenure and car ownership are available, though these may also reflect different cultural patterns between countries rather than deprivation *per se* (for example, the different

For peer review only

role that renting plays in the German housing market[39]). Some studies have also questioned whether car ownership is a good indicator of deprivation[40,41]. Measures of unemployment might also be challenged as not fully comparable either, due to the large-scale diversion of working-age adults into economic inactivity (e.g. disability benefits) during the 1990s across many European countries[42]. Exploring options to overcome these methodological challenges might be a useful avenue for future research.

Data restrictions mean we were unable to explore systematically the degree of social segregation or migration within each region. Spatial inequalities observed could simply reflect greater population heterogeneity between districts within each region. However, evidence comparing WCS with the Ruhr and Nord-Pas-de-Calais does not support this hypothesis[43,44]. Nor can we say how spatial inequalities in LE changed within these regions over time, since the analysis is also confined to a single time period. Lack of individual-level data and common markers of socio-economic status meant this study was also confined to a focus on spatial differences in life expectancy. If data had been available, analysis by inequalities by socio-economic status or other characteristics (e.g. rurality, ethnicity) may have led to different conclusions. For example, in Northern Moravia the gap in male life expectancy between districts was approximately 5 years[45] but the gap in life expectancy between the highest- and least-educated males has been enumerated at16.5 years[46].

The more pronounced spatial inequalities in life expectancy in three of the four UK regions, especially WCS, are notable. What factors might help account for this? As reported elsewhere, despite relatively high levels of mean prosperity and lower unemployment, WCS and the other British regions have higher levels of relative poverty, income inequality and

single person and lone parent households compared to post-industrial areas of mainland Europe[32]. There is also a more mixed pattern on some other indicators (e.g. social capital, educational attainment)[32]. It would be appropriate to consider the socio-political context to this. Others have contrasted the UK 'path destructive' road to deindustrialisation, characterised by the growth of a low wage service sector and reduced social protection, with alternative strategies pursued in mainland Europe[24,47]. It has been argued that a more rapid adoption of neoliberal politics by local government in WCS alongside greater vulnerability to the deleterious impacts of associated economic policies might provide some basis for explaining the findings for WCS[24, 48].

There may be differences between regions in the homogeneity of the populations, and the degree to which there is social segregation. It is possible that the greater disparities observed in WCS could be due to greater social segregation rather than larger socioeconomic inequalities (although the likelihood of this is reduced by the same finding being observed at two different sizes of sub-regional districts). The limited analyses available (comparing spatial segregation in Nord-Pas-de-Calais and Merseyside with WCS) suggests this cannot provide a wholly adequate explanation for the results shown here[31]. Nor is it clear that stronger within-region migration (from the unhealthiest to the healthiest districts) in WCS can explain these differences. One comparative study of WCS and the Ruhr (1995-2008) suggests this pattern took place in both regions and, if anything, seemed to be slightly stronger in the Ruhr than WCS[43]. This view is supported by Popham et al. (2010), who argued that selective out-migration is not the only or most important reason for the wide levels of health inequality seen in the region[49].

Differences in overall population change might provide a partial explanation. Recent long-run analysis of commune -level data for France by Ghosn et al (2012) found that population growth was associated with decreases in relative mortality[50]. Between 1982 and 2005, while most of the regions included in our study saw little change in their population, WCS saw a marked decline; while Saxony saw an even larger loss of its population over a shorter time frame[30]. This might explain why inequalities in life expectancy were wider in the Scottish region, but the much narrower inequalities in Saxony suggest that this may not be the whole story.

It may be that in other countries, 'protective' factors such as lower levels of income inequality (Northern Moravia)[51], higher levels of social capital (The Ruhr)[43] or fewer lone parent or single person households (Nord Pas de Calais)[44], or a more managed deindustrialisation process which included active labour market policies and re-employment in new industrial sectors[24], might have partly mitigated against the health-damaging effects of deindustrialisation, reducing the extent of spatial inequalities in health. However, as yet unexplained region-specific factors are also likely to play a role. Within the UK, Swansea and South Wales has relatively narrow spatial inequalities in health and WCS has some of the widest. In the former case, this may partly reflect the more homogenous social mix across ex-mining areas/villages, compared to more metropolitan areas.

Difference in lifestyle factors (i.e. worse health behaviours in WCS) could also play a role. This argument is more plausible for alcohol, since levels of consumption and alcohol-related harm are high in WCS for both genders compared to the other regions[32]. For smoking and diet, matters are less clear. Female smoking rates are higher in West Central Scotland

For peer review only

compared to most regions but male smoking rates are similar across all regions[32]. Dietary indicators suggest WCS compares poorly with Nord-Pas-de-Calais but is very similar to Merseyside and Northern Ireland[31]. That said, any explanation based on health behaviours alone would be insufficient, as the underlying causes of these health behaviours would remain unexplained.

Finally, environmental factors, such as air pollution and climate, have also been proposed as possible explanations for health inequalities. Could these factors explain the results? Richardson et al. (2013) found that while exposure to particulate air pollution (PM10), and risk of some causes of mortality, was higher in low-income European regions, but their mapping also revealed the concentration of the worst areas of pollution in East European regions (including Silesia and Northern Moravia)[52]. Although vitamin D deficiency (linked to lower levels of sunlight) may be higher in West Central Scotland than some other regions, the detrimental impacts on health are likely to be observed among older people[53]. Decomposition of the excess mortality observed in WCS compared to European regions show it to be greatest among the working-age population, especially young males and middle-aged females[30]. It therefore seems less plausible that the observed difference in spatial inequalities can be attributed to environmental factors.

CONCLUSIONS

Sub-regional spatial inequalities in life expectancy in West Central Scotland are wide compared to other post-industrial European regions, even after accounting for differences in the population size of the sub-regional districts. These spatial inequalities are particularly profound for men. By contrast, within-region spatial inequalities in life expectancy were relatively low in the German and Czech regions. These data generally show similar patterns

For peer review only

to that for inequalities by individual educational attainment for the parent countries[54]. Outside the UK, wider determinants of health (such as income distribution, positive social capital and family networks) may have acted to protect health in post-industrial regions. Future research could explore the contribution of these wider determinants of health to reducing spatial inequalities in mortality, especially in West Central Scotland.

For peer review only

ACKNOWLEDGEMENTS

This project would not have been possible without the cooperation and assistance of a number of individuals and organisations. In particular, we would like to thank the following for all their help in providing the required data:

- National Records for Scotland (NRS), formerly General Register Office for Scotland.
- Northern Ireland Statistics and Research Agency.
- Office for National Statistics and Public Health Intelligence Team, Information & Intelligence Services, Liverpool Primary Care Trust.
- Office for National Statistics (Vital Statistics).
- INSEE and Centre d'épidémiologie sur les causes médicales de décès (CepiDc) (Orignal data provided by Observatoire Régional de la Santé (ORS), NPdC).
- SPMA (https://www.wiv-isp.be/epidemio/spma), Public Health and Surveillance, Scientific Institute of Public Health, Brussels, Belgium.
- NRW Institute of Health and Work (LIGA.NRW).
- Statistisches Landesamt des Freistaates Sachsen.
- Czech Statistical Office.
- Central Statistical Office of Poland – Local Data Bank.

All underlying data used remains © to the relevant agencies.

Please note that the mortality and population data for Saxony are © Statistisches Landesamt des Freistaates Sachsen, Kamenz, 2007.

The authors would like to thank the reviewers, Peter Congdon and Paul Norman, for their useful comments on an earlier draft of this paper.

CONTRIBUTORSHIP STATEMENT

GM and DW conceived the idea for the paper and designed the study. All authors were involved in the acquisition of data, its analysis and interpretation. All authors contributed to the drafting and revising of the paper.

CONFLICTS OF INTEREST

None declared. Gerry McCartney is a member of the Scottish Socialist Party.

DATA SHARING STATEMENT: The data used to create Figures 1 and 2 are available on request from the corresponding author

For peer review only

Figure Legends

Figure 1: Inter-quartile range of life expectancy for small districts within seven post-industrial European regions, by gender, mid-to-late 2000s.

Figure 2: Inter-quartile range of life expectancy for large districts within five post-industrial European regions, by gender, mid-to-late 2000s.

For peer review only

REFERENCES

1. Scottish Government. Equally Well: Report of the Ministerial Task Force on Health Inequalities. Edinburgh: The Scottish Government; 2008.

2. Mackenbach JP. Health Inequalities: Europe in Profile. Rotterdam: Erasmus MC; 2006.

3. Shaw M, Thomas B, Davey Smith G, et al. The Grim Reaper's road map: An atlas of mortality in Britain. Bristol: The Policy Press; 2008.

4. Bonneux LG, Huisman CC, de Beer JA. Mortality in 272 European regions, 2002-2004. An update. European Journal of Epidemiology 2010;25(2):77-85.

5. Tyner JA. Population geography II: Mortality, premature death, and the ordering of life. Prog Hum Geogr, 1–14: DOI: 10.1177/0309132514527037

6. Ezzati M, Friedman AB, Kulkarni SC, et al. The reversal of fortunes: Trends in county mortality and cross-county mortality disparities in the United States. PLoS Med 2008;5(4):557-568.

7. European Commission. Health statistics – Atlas on mortality in the European Union. Luxembourg: Office for Official Publications of the European Communities; 2009.

8. Suzuki E, Kashima S, Kawachi I, et al. Social and geographic inequalities in premature adult mortality in Japan: a multilevel observational study from 1970 to 2005. BMJ Open 2012;2:e000425 doi:10 1136/bmjopen-2011-000425 .

9. Windenberger F, Rican S, Jougla E, et al. Spatiotemporal association between deprivation and mortality: trends in France during the nineties Eur J Public Health 2012;22(3):347-353.

10. Statistics Sweden. Demographic reports 2007:1, Life expectancy in Sweden 2001–2005. Life expectancy tables for the country and by county. Stockholm: Statistics Sweden; 2007.

11. Australian Bureau of Statistics. Feature Article - Regional mortality. 2002; Available at: http://www.abs.gov.au/ausstats/abs@.nsf/featurearticlesbytitle/5D0B63A4CED5187DCA256C8C007DF565?OpenDocument. Accessed August 23, 2012.

12. GUS. Life Expectancy Tables of Poland 2010. Warsaw: GUS; 2011.

13. Vallin J, Mesle F, Valkonen T. Trends in mortality and differential mortality. Strasbourg: Council of Europe; 2001.

14. Murray CJL, Michaud CM, McKenna MT et al. Patterns of Mortality by County and Race: 1965-94. Cambridge MA: Harvard Center for Population and Development Studies; 1998.

15. Office for National Statistics. Life expectancy at birth and at age 65 by local areas in the United Kingdom, 2004–06 to 2008–10. Newport: ONS.

16. Edenharter G, Haas K, Schober-Halstenberg HJ, et al. Regional Life Expectancy in Germany: Life Table Analysis for 1997/1999 to 2004/2006. Part A: Methods and Summary of Results. 2009; Available at: http://www.edenharter-research.de/regle_de/pdf/RegLE_DE_PartA.pdf. Accessed August 23, 2012.

For peer review only

17. Hacking JM, Muller S, Buchan IE, Trends in mortality from 1965 to 2008 across the English north-south divide: comparative observational study. *BMJ* 2011;342:d508

18. Dorling D. Death in Britain: how local mortality rates have changed: 1950s - 1990s. York: Joseph Rowntree Foundation.; 1997.

19. Jackson AL, Davies CA, Leyland AH. Do differences in the administrative structure of populations confound comparisons of geographic health inequalities? BMC Medical Research Methodology 2010;10:74.

20. Rees P, Brown D, Norman P, et al.Are socioeconomic inequalities in mortality decreasing or increasing within some British regions? An observational study, 1990–1998. *Journal of Public Health Medicine*, 2003; 25(3): 208–214.

21. Riva M, Terashima M, Curtis S, et al. Coalfield health effects: variation in health across former coal field areas in England. Health Place 2011;17:588e97.

22. Riva M, Curtis SE. Long-term local area employment rates as predictors of individual mortality and morbidity: a prospective study in England, spanning more than two decades. J Epidemiol Community Health 2012, 66, 919-926.

23. Bartley M. Unemployment and ill health: understanding the relationship. J Epidemiol Community Health 1994; 48:333e7.

24. Collins C, McCartney G. The impact of neoliberal 'political attack' on health: the case of the 'Scottish Effect'. Int J Health Services 2011;41(3):501-513.

25. Application of cluster analysis towards the development of health region peer groups. Statistical Society of Canada, Annual Meeting; June 2003; 2003.

For peer review only

26. Shaw M, Orford S, Brimblecombe N, et al. Widening inequality in mortality between 160 regions of 15 European countries in the early 1990s. Social Science & Medicine ;50:1047-1058.

27. Whyte B. Scottish mortality in a European context 1950 - 2000 An analysis of comparative mortality trends. Edinburgh: Information Services, NSS; 2006.

28. McCartney G, Walsh D, Whyte B, et al. Has Scotland always been the 'sick man' of Europe? An observational study from 1855 to 2006. Eur J Public Health 2011.

29. Leyland A. Increasing inequalities in premature mortality in Great Britain. J Epidemiol Community Health 2004;58:296-302.

30. Walsh D, Taulbut M, Hanlon P. The aftershock of deindustrialization—trends in mortality in Scotland and other parts of post-industrial Europe. Eur J Public Health 2010;20:58-64.

31. Taulbut M, Walsh D, Parcell S, et al. Health and its determinants in Scotland and other parts of post-industrial Europe: The Aftershock of Deindustrialisation Study phase two. Glasgow: GCPH; 2011.

32. Taulbut M, Walsh D, Parcell S, et al. What can ecological data tell us about reasons for divergence in health status between West Central Scotland and other regions of post-industrial Europe?, Public Health; 2012http://dx.doi.org/10.1016/j.puhe.2012.11.007

33. Chiang CL. The Life Table and Mortality Analysis. Geneva: World Health Organization; 1978.

34. Openshaw S. The Modifiable Areal Unit Problem. Norwich: Geo Books; 1984.

For peer review only

35. Hoffmann et al. Social differences in avoidable mortality between small areas of 15 European cities: an ecological studyInternational Journal of Health Geographics 2014, 13:8http://www.ij-healthgeographics.com/content/13/1/8

36. Thomas B, Dorling D, Davey Smith G. Inequalities in premature mortality in Britain: observational study from 1921 to 2007. *BMJ* 2010;341:c3639

37. Munoz-Arroyo R, Sutton M. Measuring Socio-Economic Inequalities in Health: A Practical Guide. Edinburgh: ScotPHO; 2007.

38. Rose D, Harrison E. The European Socio-Economic Classification European Societies, 2007 9(3); 459-490

39. Whitehead CME, Scanlon K. Social housing in Europe. In: Whitehead CM E, Scanlon K. (eds.) Social Housing in Europe. LSE London, London, pp. 8-33.

40. Focas, C. The four world cities transport study. London: The Stationery Office; 1998.

41. Johnson V., Currie G., Stanley J. Measures of Disadvantage: is Car Ownership a Good Indicator? Soc Indic Res (2010) 97:439–450

42. OECD (2003) Transforming Disability into Ability: Policies to Promote Work and Income Security for Disabled People. Paris: OECD.

43. Parcell S, Hartmann A, Taulbut M, et al. Case study: Health and its determinants in West Central Scotland compared to the Ruhr area in Germany. Glasgow: GCPH; 2011.

44. Taulbut M, Poirier G, Walsh D. Case study: Health and its determinants in West Central Scotland compared to Nord-Pas-de-Calais in France. Glasgow: GCPH; 2011.

For peer review only

45. CZSO. Life expectancy in districts (LAU 1) in 2005-2009 (data extra regular outputs). Available at: http://www.czso.cz/eng/redakce.nsf/i/life_tables. Accessed August

46. van Raalte AA, Kunst AE, Lundberg O, et al. The contribution of educational inequalities to lifespan variation. Population Health Metrics 2012;10(3).

47. Birch K, Mykhnenko K. Varieties of neoliberalism? Restructuring in large industrially dependent regions across Western and Eastern Europe. Journal of Economic Geography 2009; 9: 355–380.

48. Boyle M, McWilliams C, Rice G. The spatialities of actually existing neoliberalism in Glasgow, 1977 to present. 2008;90(4):313-325.

49. Popham F, Boyle PJ, O'Reilly D, et al. Selective internal migration. Does it explain Glasgow's worsening mortality record? Health Place. 2011 Nov;17(6):1212-7. doi: 10.1016/j.healthplace.2011.08.004. Epub 2011 Aug 22.

50. Ghosn W, Kassié D, Jougla1 E, et al. Trends in geographic mortality inequalities and their association with population changes in France, 1975–2006. European Journal of Public, 23(5), 834-840.

51. Walsh D, Strniskova D, Taulbut M. Case study: Health and its determinants in West Central Scotland compared to Northern Moravia in the Czech Republic. Glasgow: GCPH; 2011.

52. Richardson EA, Pearce J, Tunstall H, et al. Particulate air pollution and health inequalities: a Europe-wide ecological analysis. International Journal of Health Geographics 2013, 12:34

53. Rush L, McCartney G, Walsh D, et al. Vitamin D and subsequent all-age and premature mortality: a systematic review. BMC Public Health 2013; 13: 679.

For peer review only

54. Popham F, Boyle P. Assessing socio-economic inequalities in mortality and other health outcomes at the Scottish national level. Edinburgh: Scottish Collaboration for Public Health Research and Policy; 2011.

For peer review only

Spatial inequalities in life expectancy within post-industrial regions of Europe: a cross-sectional observational study

Formatted: Font: 14 pt

Formatted: Font: 14 pt, Bold

Formatted: Font: 14 pt

Martin Taulbut[1], David Walsh[2], Gerry McCartney[3], Sophie Parcell[4], Anja Hartmann[5], Gilles Poirier[6], Dana Strniskova[7], Phil Hanlon[8].

NHS Health Scotland

Meridian Court

5 Cadogan Street

Glasgow G2 6QE.

Scotland.

Tel: +44 0141 414 2754

Correspondence to: martintaulbut@nhs.net

1. NHS Health Scotland, Glasgow, Scotland.
2. Glasgow Centre for Population Health, Glasgow, Scotland.
3. NHS Health Scotland, Glasgow, Scotland.
4. Glasgow Centre for Population Health, Glasgow, Scotland.
5. Ruhr-Universität Bochum Germany.
6. Observatoire Régional de la Santé (ORS), Nord-Pas-de-Calais.
7. Regional Public Health Authority of the Olomouc Region of the Czech Republic.
8. University of Glasgow, Glasgow, Scotland.

3,500 words

For peer review only

Abstract

Objectives To compare spatial inequalities in life expectancy in West Central Scotland (WCS) with nine other post-industrial European regions.

Design A cross-sectional observational study.

Setting West Central Scotland and nine other post-industrial regions across Europe.

Participants Data for WCS and nine other, comparably deindustrialised, European regions were analysed. Male and female life expectancies (LE) at birth were obtained or calculated for the mid-2000s for 160 districts within selected regions. Districts were stratified into two groups: small (populations of between 141,000 and 185,000 people) and large (populations between 224,000 and 352,000). The range and inter-quartile range (IQR) in LE were used to describe within-region disparities.

Results In small districts, the male LE range was widest in WCS and Merseyside, while the IQR was widest in WCS and Northern Ireland. For women, the LE range was widest in WCS, though the IQR was widest in Northern Ireland and Merseyside. In large districts, the range and IQR in life expectancy was widest in WCS and Wallonia for both sexes.

Conclusions Sub-regional spatial inequalities in life expectancy in WCS are wide compared to other post-industrial mainland European regions, especially for men. Future research could explore the contribution of economic, social and political factors in reducing these inequalities.

For peer review only

Strengths and limitations of this study

- This is an extensive, international comparison of contemporary, within-region disparities in life expectancy. It compares 100 small districts and 60 large districts across 10 European regions.
- Ecological bias was mitigated by selecting regions with a similar history of deindustrialisation and comparing districts with similar–sized populations.
- While the approach taken here partly addressed the scale issue associated with the 'Modifiable Area Unit Problem', it was unable to resolve the zoning issue.
- The study was unable to say whether more heterogeneous populations or higher levels of social segregation were driving these differences, though the limited evidence we have does not support this view.
- The analyses are restricted to one period during the mid-to-late 2000s.
- The approach was restricted to describing spatial differences in life expectancy – we cannot draw any conclusions on within-region inequalities by socio-economic status, rurality or ethnicity.

For peer review only

INTRODUCTION

Reducing inequalities in health has been identified as a priority by governments across Europe[1,2]. While inequalities in health are often described using individual characteristics (e.g. socio-economic class), there is also considerable interest in spatial disparities in health[3,4], despite a lack of research found by Tyner et al. (2014)[5]. All countries exhibit sub-national variation in mortality and life expectancy[5,6,76,7,8]. The pattern is observed for countries as diverse as France[98], Sweden[9]Sweden[10], Australia[10] Australia[11] and Poland[11]Poland[12]. Almost universally, the geographical gap in these health outcomes is wider for men than women[132]. There are some observed differences in within-country dispersion in life expectancy, with the spatial gap more pronounced for some nations (e.g. USA[143], UK[154]) than others (e.g. Germany[165], Poland[121]). Regional inequalities in mortality between English regions, for instance, have been found to be severe and persistent over a forty-year period[17]. Differences are also observed in whether spatial inequality in mortality has been narrowing, static or increasing over time[186,132]., Although findings are dependent on the size of geographies selected for analysis[197,], there is evidence that inequalities both between and within English regions have increased over time[17,20].,

Formatted: Superscript

Formatted: Superscript

Formatted: Superscript

Deindustrialisation has been proposed as a mechanism to partly explain these spatial inequalities. Across Europe, there is a clear overlap between former coalmining and industrial areas and districts and regions with the poorest health[18]health[21,76]. A recent study in England Riva et al. (2012) found that areas in England with persistently low or deteriorating employment rates (relative to the national average), often located in ex-

For peer review only

industrial regions, had the highest rates of mortality and physical morbidity, even after adjusting for migration and individual characteristics of residents[2219]. A number of mechanisms (e.g. greater poverty, loss of purpose and status and higher levels of substance misuse) provide plausible links between economic dislocation and health outcomes[230, 241].

Making spatial comparisons of health within and between geographies is subject to a number of difficulties. Comparing geographies that have been ‘clustered’ according to some shared characteristics (such as a similar economic and social history) can partly adjust for this and produce more meaningful results[252]. Geographical comparisons are more valid when the spatial units being compared are of a similar population size and where there is less social diversity within them, since the differences between areas will depend on the degree to which the geographical units of analysis are internally diverse or homogeneous. Units of analysis with larger population sizes or more heterogeneity in their composition are less likely to display differences between areas because of the averaging effect of this greater internal diversity[263,197]. Failing to take this into account may result in misleading comparisons.

The present study approaches this issue from a Scottish perspective. Scotland’s position as the ‘sick man’ of Europe – characterised by a slower rate of improvement in life expectancy compared to other West European nations since the 1950s, and a consequent relative deterioration in its international position – has been discussed elsewhere[274,285]. Furthermore, the within-region spatial gap in mortality was greater in Scotland than any other region of Britain[296]. A similar ‘faltering’ in the pace of improvement in mortality and life expectancy has also been noted for West Central Scotland (WCS), the region of Scotland most affected by deindustrialisation in recent decades, relative to other post-industrial regions[3027]. Post-

For peer review only

industrial regions are extremely important in epidemiological terms as they tend to exhibit the highest rates of mortality in their parent countries~~[28,29]~~[31,32]. A more recent study also suggested that WCS was more spatially divided in terms of mortality than other, comparable European post-industrial regions, though the authors did not pursues this question in ~~depth[28]~~depth[31]. This paper explores this question in a systematic way, to investigate whether spatial disparities in mortality within WCS are large compared to other European regions, taking both industrial heritage and differences in population sizes of sub-regions into account.

METHODS

This study was informed by the authors' involvement in a larger project which aimed to contribute to an understanding of the poor health observed in one post-industrial region, West Central Scotland, in the context of other comparable European regions. West Central Scotland (WCS) is a region of 2.1 million people, centred on the City of Glasgow. Nine other regions, highlighted in other recent epidemiological ~~analysis[27]~~analysis[30,32]~~[29]~~, were selected for comparison with WCS. The regions were chosen through consultation with experts on European history on the basis of their shared historic economic dependence on industries such as coal, steel, shipbuilding and textiles, alongside analysis of their subsequent loss of industrial employment over the last 30-40 ~~years[27]~~years[30].

Table 1 presents summary information on the list of regions selected. Selecting a range of regions from across East and West Europe allowed contrasts to be made between WCS and European areas with different social and political context. The inclusion of UK regions

For peer review only

meant the WCS could be compared with areas subject to the same set of socio-economic policies over the last 30-40 years.

For peer review only

Table 1

Region name	Nation state	Number of districts	Mean population size of districts	Principal historical industries	Total industrial employment loss[a]
West Central Scotland	UK	15[b] (7)[c]	141,268[b] (302,084)[c]	Shipbuilding and support industries (iron, coal, engineering)	-62% (1971-2005)
Northern Ireland	UK	12	147,900	Shipbuilding, textiles, manufacturing	-20% (1971-2005)
Merseyside	UK	9	149,532	Shipping, docks, manufacturing (e.g. cement), engineering	-63% (1971-2005)
Swansea & S. Wales Coalfields	UK	7	160,486	Coal	-51% (1971-2005)
Nord-Pas-de-Calais	~~UK~~France	25	160,746	Coal, textiles, steel	-43% (1970-2005)
Wallonia	Belgium	11	309,542	Mining, metal working, textiles	-39% (1970-2005)
The Ruhr	Germany	15	351,912	Coal, iron, steel	-54% (1970-2005)
Saxony	Germany	19	224,934	Steel, construction, engineering, textiles	-47% (1991-2005)
Northern Moravia	Czech Republic	11[d]	185,099	Coal, steel	-19% (1993-2005)
Silesia[e]	Poland	29	159,858	Coal, steel, automobiles, zinc	-55% (1980-2005)

a Percentage decrease in the number of industrial jobs in each region over the time period shown in parenthesis. For Silesia, change is shown for the Katowice sub-region.

b Community Health Partnerships.

c Nomenclature of Units for Territorial Statistics (NUTS) 3.

d Jesenik district included in small district comparisons only.

e Known as Slaskie region in Poland.

For peer review only

Male and female life expectancies at birth were obtained from relevant statistical agencies (or where appropriate calculated) for the mid-2000s, for 160 districts within the 10 selected regions. Ideally, the years of the data collected would be of identical time frame and size. It was not possible or practical to do so here, because of variation between countries in terms of availability of the required small area statistics data. All life tables were constructed in the same way, using all deaths within each district and the resident population of each district. The sources of the life expectancy data for each region are given in Table 2 (web-only table).

In order to reduce the risk of bias due to differing sub-regional population sizes (the scale problem), we stratified the regions into two. Five regions (Swansea & S. Wales Coalfields, N. Ireland, Nord-Pas-de-Calais, Silesia and Merseyside) had sub-regional (or district) populations of between 141,000 and 185,000 people. These areas were compared to similarly-sized geographies in WCS Community Health Partnership areas (CHPs).[1] Three regions (the Ruhr, Saxony and Wallonia) had life expectancy data calculated across 45 'large' districts of population size ranging from 224,000 and 352,000: these were compared with similarly-sized WCS Nomenclature of Units for Territorial Statistics (NUTS) 3 areas. Data for Northern Moravia and WCS were available for both strata. For four regions (Northern Ireland, Wallonia, Silesia and Nord-Pas-de-Calais), it was necessary to create pseudo-districts to ensure a more even distribution of population across districts. This process took into account contiguous boundaries and where possible the character of districts. Life expectancy at birth was then calculated for these new areas using the Chiang method (II)[330], using population and mortality data obtained from the relevant national statistical agencies.

[1] There were 15 CHP areas in WCS prior to April 2010, when the five Glasgow CHPs were merged into three.

For peer review only

Within regions, we then ranked the sub-regional (district) populations by their life expectancy separately for men and women and separately for the large and small sub-regional populations. We then created line graphs for each strata of regions to show the size and distribution of sub-regional populations and their corresponding life expectancies. Taking each region separately, we then calculated the range in life expectancy and inter-quartile ranges, accounting for the population sizes in each sub-regional district, to describe the within-regional disparities.

For peer review only

RESULTS

Regions with small district data (populations between 141,000 and 185,000)

The districts with the highest male life expectancies (>77 years at birth) were in the rural districts in Northern Ireland, plus the more affluent WCS districts of East Renfrewshire and East Dunbartonshire. The lowest male life expectancies (<70 years at birth) were in Silesia and in areas of WCS (North and East Glasgow). The districts with the highest levels of female life expectancy (>82.5 years at birth) were all located in Nord-Pas-de Calais whilst the lowest (<78 years at birth) were in WCS (all five Glasgow districts), Merseyside (City & North Liverpool) and parts of the Silesia region (Ruda Slaska & Swietochlowice and Chorzow & Siemianowice Slaskie).

Within regions, the *range* in male life expectancy was widest for WCS (8.6 years) and Merseyside (5.9 years) and narrowest in Swansea & the South Wales Coalfields (1.6 years) and Northern Moravia (2.7 years). The *inter-quartile range* in life expectancy for men was widest in WCS and Northern Ireland (2.7 years and 2.6 years respectively) followed by Silesia (2.2 years) and was much less pronounced in the other regions. For women, WCS had the widest *range* in life expectancy (6.5 years) and Northern Moravia the narrowest (1.6 years). The range of life expectancies observed for Merseyside districts was also high (5.9). The *inter-quartile range* in female life expectancy was highest in Northern Ireland (2.0 years) and Merseyside (1.9 years) and lowest in Northern Moravia (Figure 1).

For peer review only

[Figure 1 about here]

Regions with large district data (populations between 224,000 and 352,000)

The highest male life expectancies were found in Saxony, Wallonia and the Ruhr, whilst the lowest were observed in WCS (Glasgow), Wallonia (Mons) and in Northern Moravia. For women, districts with the highest life expectancy were located in Wallonia and Saxony, while the districts with the lowest life expectancy were found within WCS and Northern Moravia.

Within regions, the *range* in male life expectancy across 'large' districts was widest for WCS (5.3 years) followed by Wallonia (4.8 years), with the Ruhr Valley, Saxony and Northern Moravia less polarised. The *inter-quartile range* in life expectancy was much wider in WCS (3.9 years) than in all other regions. For women, the pattern was similar, with the widest range in life expectancy observed for WCS (3.5 years) and Wallonia (2.5 years), with much less disparity evident in the German and Czech regions (Figure 2).

[Figure 2 about here]

For peer review only

DISCUSSION

Similarly deindustrialised regions in Europe, which share similar economic, social and health ~~problems~~[27] problems[30,32][29], display different patterns in spatial inequalities in life expectancy. In particular, two UK regions (WCS and Merseyside) have much larger intra-regional differences in life expectancy for both men and women than the other regions, with WCS having the largest differences. In contrast, there are relatively narrow spatial inequalities in life expectancy in Northern Moravia, the Ruhr and Swansea and South Wales Coalfields.

The present study has four important strengths. First, it provides an original comparison of contemporary, international, within-region disparities in life expectancy. Second, its geographical coverage is extensive: more than 100 small districts and 60 large districts, spanning 10 regions across both Western and Eastern Europe. Third, it uses a straightforward metric of health outcomes (life expectancy at birth) that is readily understood. Finally, by attempting to ensure the areas are of a similar size and have a common experience of industrial development and subsequent deindustrialisation, the potential bias arising from comparisons of differently sized populations and the heterogeneity within regions is reduced.

The study also has a number of limitations. A key challenge in any study of this kind is the 'Modifiable Area Unit Problem' (MAUP). As discussed by Openshaw (1984)[341], the spatial units that can be used to describe individual level data are usually highly modifiable and their boundaries are often decided on an arbitrary basis. There are a large number of different spatial units that could be used to describe the same data, often producing quite different conclusions. There are two components of the MAUP. First, there is a scale problem, with

For peer review only

different results being produced depending on the number of spatial units used in analysis (e.g. for Census Tracts, districts, regions). Second, there is a grouping or zoning problem, reflecting different choices about how very small areas are joined together to create areas of a similar size. In this study, the scale problem has been partly addressed by making comparisons of sub-regional inequalities at two different geographical levels. The similar findings (of greater spatial inequalities in WCS) for both scales can give more confidence that the approach adopted is reasonable. However, the zoning problem remains difficult to resolve without access to individual-level data coded to geographic areas, which are currently not available. It is important to note that the findings may not apply beyond the selection of post-industrial regions shown here. For example, Hoffman et (2012), who analysed neighbourhood-level differences in mortality for 15 large European large cities, found that inequalities were wider for women than for men, and no evidence that within-area inequalities varied between cities[35].

Formatted: Superscript

The methods used to compare spatial inequalities (interquartile range) could also be criticised as not ideal. Other studies[36] have used the slope index of inequality (SII) and relative index of inequality (RII) to estimate spatial inequalities in mortality[37]. This would undoubtedly allow for more robust analyses. However, to allow these indices to be constructed would require robust, internationally comparable measures for ranking all the districts by socio-economic status. Data limitations make this a difficult task. Europe-wide indicators of material and income deprivation are unavailable for small-area geographies. A prototype European Socio-economic Classification[38] has been developed but comparable small area data (from national Censuses) for all areas are not yet available. Limited measures of housing tenure and car ownership are available, though these may also reflect different cultural patterns between countries rather than deprivation *per se* (for example, the different

Formatted: Superscript

Formatted: Superscript

Formatted: Superscript

For peer review only

role that renting plays in the German housing market[39]). Some studies have also questioned whether car ownership is a good indicator of deprivation[40,41]. Measures of unemployment might also be challenged as not fully comparable either, due to the large-scale diversion of working-age adults into economic inactivity (e.g. disability benefits) during the 1990s across many European countries[42]. Exploring options to overcome these methodological challenges might be a useful avenue for future research.

Formatted: Superscript

Formatted: Superscript

Formatted: Superscript

Data restrictions mean we were unable to explore systematically the degree of social segregation or migration within each region. Spatial inequalities observed could simply reflect greater population heterogeneity between districts within each region. However, evidence comparing WCS with the Ruhr and Nord-Pas-de-Calais does not support this hypothesis32,33[43,44]. Nor can we say how spatial inequalities in LE changed within these regions over time, since the analysis is also confined to a single time period. Lack of individual-level data and common markers of socio-economic status meant this study was also confined to a focus on spatial differences in life expectancy. -If data had been available, analysis by inequalities by socio-economic status or other characteristics (e.g. rurality, ethnicity) may have led to different conclusions. For example, in Northern Moravia the gap in male life expectancy between districts was approximately 5 years34 years[45] but the gap in life expectancy between the highest- and least-educated males has been enumerated at16.5 years35years[46].

Formatted: Not Superscript/ Subscript

Formatted: Superscript

Formatted: Not Superscript/ Subscript

Formatted: Superscript

Formatted: Not Superscript/ Subscript

Formatted: Superscript

For peer review only

The more pronounced spatial inequalities in life expectancy in three of the four UK regions, especially WCS, are notable. What factors might help account for this? As reported elsewhere, despite relatively high levels of mean prosperity and lower unemployment, WCS and the other British regions have higher levels of relative poverty, income inequality and single person and lone parent households compared to post-industrial areas of mainland ~~Europe~~[29]Europe[32]. There is also a more mixed pattern on some other indicators (e.g. social capital, educational attainment)[2932]. It would be appropriate to consider the socio-political context to this. Others have contrasted the UK ‘path destructive’ road to deindustrialisation, characterised by the growth of a low wage service sector and reduced social protection, with alternative strategies pursued in mainland Europe[241,3647]. It has been argued that a more rapid adoption of neoliberal politics by local government in WCS alongside greater vulnerability to the deleterious impacts of associated economic policies might provide some basis for explaining the findings for WCS[241, 48 37].

Formatted: Not Superscript/ Subscript

There may be differences between regions in the homogeneity of the populations, and the degree to which there is social segregation. It is possible that the greater disparities observed in WCS could be due to greater social segregation rather than larger socioeconomic inequalities (although the likelihood of this is reduced by the same finding being observed at two different sizes of sub-regional districts). The limited analyses available (comparing spatial segregation in Nord-Pas-de-Calais and Merseyside with WCS) suggests this cannot provide a wholly adequate explanation for the results shown here[31]. Nor is it clear that stronger within-region migration (from the unhealthiest to the healthiest districts) in WCS can explain these differences. One comparative study of WCS and the Ruhr (1995-2008)

Formatted: Font color: Auto

suggests this pattern took place in both regions and, if anything, seemed to be slightly stronger in the Ruhr than WCS[43]. This view is supported by Popham et al. (2010), who argued that selective out-migration is not the only or most important reason for the wide levels of health inequality seen in the region[49].

Formatted: Superscript

Differences in overall population change might provide a partial explanation. Recent long-run analysis of commune -level data for France by Ghosn et al (2012) found that population growth was associated with decreases in relative mortality[50]. Between 1982 and 2005, while most of the regions included in our study saw little change in their population, WCS saw a marked decline; while Saxony saw an even larger loss of its population over a shorter time frame[30]. This might explain why inequalities in life expectancy were wider in the Scottish region, but the much narrower inequalities in Saxony suggest that this may not be the whole story.

Formatted: Superscript

It may be that in other countries, ‘protective’ factors such as lower levels of income inequality (Northern Moravia)[5138], higher levels of social capital (The Ruhr)[32-43] or fewer lone parent or single person households (Nord Pas de Calais)[3344], or a more managed deindustrialisation process which included active labour market policies and re-employment in new industrial sectors[241], might have partly mitigated against the health-damaging effects of deindustrialisation, reducing the extent of spatial inequalities in health. However, as yet unexplained region-specific factors are also likely to play a role. Within the UK, Swansea and South Wales has relatively narrow spatial inequalities in health and WCS has some of the widest. In the former case, this may partly reflect the more homogenous social mix across ex-mining areas/villages, compared to more metropolitan areas.

For peer review only

Difference in lifestyle factors (i.e. worse health behaviours in WCS) could also play a role. This argument is more plausible for alcohol, since levels of consumption and alcohol-related harm are high in WCS for both genders compared to the other regions[32][29]. For smoking and diet, matters are less clear. Female smoking rates are higher in West Central Scotland compared to most regions but male smoking rates are similar across all regions[29] regions[32]. Dietary indicators suggest WCS compares poorly with Nord-Pas-de-Calais but is very similar to Merseyside and Northern Ireland[28] Ireland[31]. That said, any explanation based on health behaviours alone would be insufficient, as the underlying causes of these health behaviours would remain unexplained.

Finally, environmental factors, such as air pollution and climate, have also been proposed as possible explanations for health inequalities. Could these factors explain the results? Richardson et al. (2013) found that while exposure to particulate air pollution (PM10), and risk of some causes of mortality, was higher in low-income European regions, but their mapping also revealed the concentration of the worst areas of pollution in East European regions (including Silesia and Northern Moravia)[52]. Although vitamin D deficiency (linked to lower levels of sunlight) may be higher in West Central Scotland than some other regions, the detrimental impacts on health are likely to be observed among older people[53]. Decomposition of the excess mortality observed in WCS compared to European regions show it to be greatest among the working-age population, especially young males and middle-aged females[30]. It therefore seems less plausible that the observed difference in spatial inequalities can be attributed to environmental factors.

Formatted: Font: Not Bold

Formatted: Font: (Default) Times New Roman, 12 pt

Formatted: Superscript

Formatted: Superscript

Formatted: Font: Not Bold

CONCLUSIONS

For peer review only

Sub-regional spatial inequalities in life expectancy in West Central Scotland are wide compared to other post-industrial European regions, even after accounting for differences in the population size of the sub-regional districts. These spatial inequalities are particularly profound for men. By contrast, within-region spatial inequalities in life expectancy were relatively low in the German and Czech regions. These data generally show similar patterns to that for inequalities by individual educational attainment for the parent countries[54][~~39~~]. Outside the UK, wider determinants of health (such as income distribution, positive social capital and family networks) may have acted to protect health in post-industrial regions. Future research could explore the contribution of these wider determinants of health to reducing spatial inequalities in mortality, especially in West Central Scotland.

ACKNOWLEDGEMENTS

This project would not have been possible without the cooperation and assistance of a number of individuals and organisations. In particular, we would like to thank the following for all their help in providing the required data:

- National Records for Scotland (NRS), formerly General Register Office for Scotland.
- Northern Ireland Statistics and Research Agency.
- Office for National Statistics and Public Health Intelligence Team, Information & Intelligence Services, Liverpool Primary Care Trust.
- Office for National Statistics (Vital Statistics).
- INSEE and Centre d'épidémiologie sur les causes médicales de décès (CepiDc) (Orignal data provided by Observatoire Régional de la Santé (ORS), NPdC).
- SPMA (https://www.wiv-isp.be/epidemio/spma), Public Health and Surveillance, Scientific Institute of Public Health, Brussels, Belgium.
- NRW Institute of Health and Work (LIGA.NRW).
- Statistisches Landesamt des Freistaates Sachsen.
- Czech Statistical Office.
- Central Statistical Office of Poland – Local Data Bank.

All underlying data used remains © to the relevant agencies.

Please note that the mortality and population data for Saxony are © Statistisches Landesamt des Freistaates Sachsen, Kamenz, 2007.

The authors would like to thank the reviewers, Peter Congdon and Paul Norman, for their useful comments on an earlier draft of this paper.

CONFLICTS OF INTEREST

None declared. Gerry McCartney is a member of the Scottish Socialist Party.

REFERENCES

1. Scottish Government. Equally Well: Report of the Ministerial Task Force on Health Inequalities. Edinburgh: The Scottish Government; 2008.

2. Mackenbach JP. Health Inequalities: Europe in Profile. Rotterdam: Erasmus MC; 2006.

3. Shaw M, Thomas B, Davey Smith G, Dorling D. The Grim Reaper's road map: An atlas of mortality in Britain. Bristol: The Policy Press; 2008.

For peer review only

4. Bonneux LG, Huisman CC, de Beer JA. Mortality in 272 European regions, 2002-2004. An update. European Journal of Epidemiology 2010;25(2):77-85.

5. Tyner JA. Population geography II: Mortality, premature death, and the ordering of life. Prog Hum Geogr, 1–14: DOI: 10.1177/0309132514527037

6. Ezzati M, Friedman AB, Kulkarni SC, Murray CJL. The reversal of fortunes: Trends in county mortality and cross-county mortality disparities in the United States. PLoS Med 2008;5(4):557-568.

7~~6~~. European Commission. Health statistics – Atlas on mortality in the European Union. Luxembourg: Office for Official Publications of the European Communities; 2009.

8~~7~~. Suzuki E, Kashima S, Kawachi I, Subramanian SV. Social and geographic inequalities in premature adult mortality in Japan: a multilevel observational study from 1970 to 2005. BMJ Open 2012;2:e000425 doi:10 1136/bmjopen-2011-000425 .

9~~8~~. Windenberger F, Rican S, Jougla E, Rey G. Spatiotemporal association between deprivation and mortality: trends in France during the nineties Eur J Public Health 2012;22(3):347-353.

10~~9~~. Statistics Sweden. Demographic reports 2007:1, Life expectancy in Sweden 2001–2005. Life expectancy tables for the country and by county. Stockholm: Statistics Sweden; 2007.

11~~0~~. Australian Bureau of Statistics. Feature Article - Regional mortality. 2002; Available at: http://www.abs.gov.au/ausstats/abs@.nsf/featurearticlesbytitle/5D0B63A4CED5187DCA256C8C007DF565?OpenDocument. Accessed August 23, 2012.

12~~1~~. GUS. Life Expectancy Tables of Poland 2010. Warsaw: GUS; 2011.

13~~2~~. Vallin J, Mesle F, Valkonen T. Trends in mortality and differential mortality. Strasbourg: Council of Europe; 2001.

For peer review only

143. Murray CJL, Michaud CM, McKenna MT, Marks JS. U.S. Patterns of Mortality by County and Race: 1965-94. Cambridge MA: Harvard Center for Population and Development Studies; 1998.

154. Office for National Statistics. Life expectancy at birth and at age 65 by local areas in the United Kingdom, 2004–06 to 2008–10. Newport: ONS.

165. Edenharter G, Haas K, Schober-Halstenberg HJ, Frei U. Regional Life Expectancy in Germany: Life Table Analysis for 1997/1999 to 2004/2006. Part A: Methods and Summary of Results. 2009; Available at: http://www.edenharter-research.de/regle_de/pdf/RegLE_DE_PartA.pdf. Accessed August 23, 2012.

176. Hacking JM, Muller S, Buchan IE, Trends in mortality from 1965 to 2008 across the English north-south divide: comparative observational study. *BMJ* 2011;342:d508

18. Dorling D. Death in Britain: how local mortality rates have changed: 1950s - 1990s. York: Joseph Rowntree Foundation.; 1997.

197. Jackson AL, Davies CA, Leyland AH. Do differences in the administrative structure of populations confound comparisons of geographic health inequalities? BMC Medical Research Methodology 2010;10:74.

20. Rees P, Brown D, Norman P, Dorling D.Are socioeconomic inequalities in mortality decreasing or increasing within some British regions? An observational study, 1990–1998. *Journal of Public Health Medicine*, 2003; 25(3): 208–214.

1821. Riva M, Terashima M, Curtis S, Shucksmith J, Carlebach S. Coalfield health effects: variation in health across former coal field areas in England. Health Place 2011;17:588e97.

1922. Riva M, Curtis SE. Long-term local area employment rates as predictors of individual mortality and morbidity: a prospective study in England, spanning more than two decades. J Epidemiol Community Health 2012, 66, 919-926.

Formatted: Font: (Default) Times New Roman, 12 pt, Italic

Formatted: Font: (Default) Times New Roman, 12 pt

Formatted: Font: Italic

For peer review only

230. Bartley M. Unemployment and ill health: understanding the relationship. J Epidemiol Community Health 1994; 48:333e7.

241. Collins C, McCartney G. The impact of neoliberal 'political attack' on health: the case of the 'Scottish Effect'. Int J Health Services 2011;41(3):501-513.

252. Application of cluster analysis towards the development of health region peer groups. Statistical Society of Canada, Annual Meeting; June 2003; 2003.

263. Shaw M, Orford S, Brimblecombe N, Dorling D. Widening inequality in mortality between 160 regions of 15 European countries in the early 1990s. Social Science & Medicine ;50:1047-1058.

274. Whyte B. Scottish mortality in a European context 1950 - 2000 An analysis of comparative mortality trends. Edinburgh: Information Services, NSS; 2006.

285. McCartney G, Walsh D, Whyte B, Collins C. Has Scotland always been the 'sick man' of Europe? An observational study from 1855 to 2006. Eur J Public Health 2011.

296. Leyland A. Increasing inequalities in premature mortality in Great Britain. J Epidemiol Community Health 2004;58:296-302.

3027. Walsh D, Taulbut M, Hanlon P. The aftershock of deindustrialization—trends in mortality in Scotland and other parts of post-industrial Europe. Eur J Public Health 2010;20:58-64.

2831. Taulbut M, Walsh D, Parcell S, et al. Health and its determinants in Scotland and other parts of post-industrial Europe: The Aftershock of Deindustrialisation Study phase two. Glasgow: GCPH; 2011.

For peer review only

~~29~~32. Taulbut M, Walsh D, Parcell S, et al. What can ecological data tell us about reasons for divergence in health status between West Central Scotland and other regions of post-industrial Europe?, Public Health; 2012http://dx.doi.org/10.1016/j.puhe.2012.11.007

33~~0~~. Chiang CL. The Life Table and Mortality Analysis. Geneva: World Health Organization; 1978.

34~~1~~. Openshaw S. The Modifiable Areal Unit Problem. Norwich: Geo Books; 1984.

35. Hoffmann et al. Social differences in avoidable mortality between small areas of 15 European cities: an ecological studyInternational Journal of Health Geographics 2014, 13:8http://www.ij-healthgeographics.com/content/13/1/8

36. Thomas B, Dorling D, Davey Smith G. Inequalities in premature mortality in Britain: observational study from 1921 to 2007. *BMJ* 2010;341:c3639

37. Munoz-Arroyo R, Sutton M. Measuring Socio-Economic Inequalities in Health: A Practical Guide. Edinburgh: ScotPHO; 2007.

38. Rose D, Harrison E. The European Socio-Economic Classification European Societies, 2007 9(3); 459-490

39. Whitehead CME, Scanlon K. Social housing in Europe. In: Whitehead CM E, Scanlon K. (eds.) Social Housing in Europe. LSE London, London, pp. 8-33.

40. Focas, C. The four world cities transport study. London: The Stationery Office; 1998.

41. Johnson V., Currie G., Stanley J. Measures of Disadvantage: is Car Ownership a Good Indicator? Soc Indic Res (2010) 97:439–450

Formatted: Font: (Default) Times New Roman, 12 pt

Formatted: Highlight

Formatted: Font: (Default) Times New Roman, 12 pt

Formatted: Font: (Default) Times New Roman, 12 pt

For peer review only

42. OECD (2003) Transforming Disability into Ability: Policies to Promote Work and Income Security for Disabled People. Paris: OECD.

~~32~~43. Parcell S, Hartmann A, Taulbut M, Walsh D. Case study: Health and its determinants in West Central Scotland compared to the Ruhr area in Germany. Glasgow: GCPH; 2011.

~~33~~44. Taulbut M, Poirier G, Walsh D. Case study: Health and its determinants in West Central Scotland compared to Nord-Pas-de-Calais in France. Glasgow: GCPH; 2011.

45~~34~~. CZSO. Life expectancy in districts (LAU 1) in 2005-2009 (data extra regular outputs). Available at: http://www.czso.cz/eng/redakce.nsf/i/life_tables. Accessed August

46~~35~~. van Raalte AA, Kunst AE, Lundberg O, Leinsalu M, Martikainen P, Artnik B, et al. The contribution of educational inequalities to lifespan variation. Population Health Metrics 2012;10(3).

~~36~~47. Birch K, Mykhnenko K. Varieties of neoliberalism? Restructuring in large industrially dependent regions across Western and Eastern Europe. Journal of Economic Geography 2009; 9: 355–380.

~~37~~48. Boyle M, McWilliams C, Rice G. The spatialities of actually existing neoliberalism in Glasgow, 1977 to present. 2008;90(4):313-325.

49. Popham F, Boyle PJ, O'Reilly D, Leyland AH. Selective internal migration. Does it explain Glasgow's worsening mortality record? Health Place. 2011 Nov;17(6):1212-7. doi: 10.1016/j.healthplace.2011.08.004. Epub 2011 Aug 22.

50~~38~~. Ghosn W, Kassié D, Jougla1 E, Salem G, Rey G, Rican S. Trends in geographic mortality inequalities and their association with population changes in France, 1975–2006. European Journal of Public, 23(5), 834-840.

For peer review only

51. Walsh D, Strniskova D, Taulbut M. Case study: Health and its determinants in West Central Scotland compared to Northern Moravia in the Czech Republic. Glasgow: GCPH; 2011.

52. Richardson EA, Pearce J, Tunstall H, Mitchell R, Shortt NM. Particulate air pollution and health inequalities: a Europe-wide ecological analysis. International Journal of Health Geographics 2013, 12:34

53. Rush L, McCartney G, Walsh D, MacKay D. Vitamin D and subsequent all-age and premature mortality: a systematic review. BMC Public Health 2013; 13: 679.

~~39~~54. Popham F, Boyle P. Assessing socio-economic inequalities in mortality and other health outcomes at the Scottish national level. Edinburgh: Scottish Collaboration for Public Health Research and Policy; 2011.

Formatted: Font: Times New Roman, 12 pt

Figure Legends

Formatted: Font: Times New Roman, 12 pt, Bold

Figure 1: Inter-quartile range of life expectancy for small districts within seven post-industrial European regions, by gender, mid-to-late 2000s.

Formatted: Font: Times New Roman, 12 pt, Italic

Formatted: Font: Italic

Figure 2: Inter-quartile range of life expectancy for large districts within five post-industrial European regions, by gender, mid-to-late 2000s.

Formatted: Font: Times New Roman, 12 pt, Italic

Formatted: Font: Italic

For peer review only

For peer review only

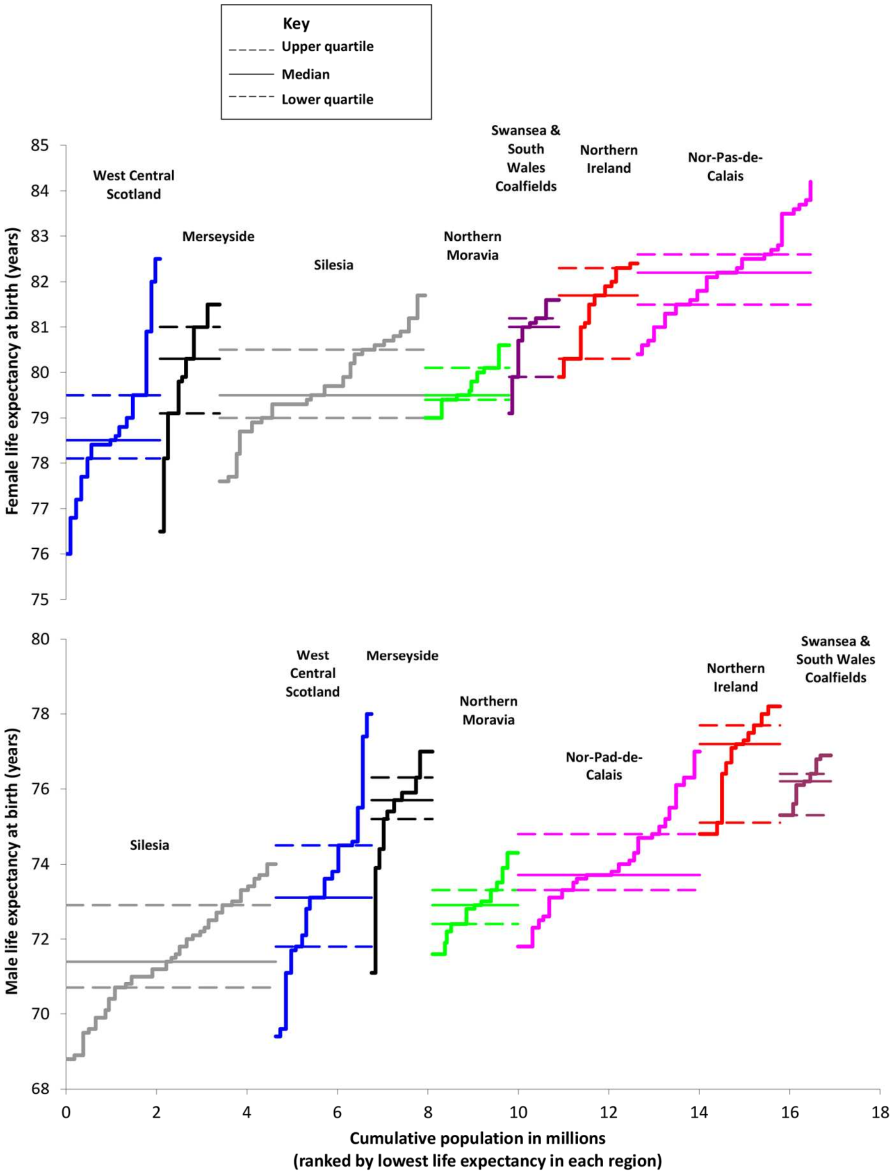

Figure 1: Inter-quartile range of life expectancy for small districts within seven post-industrial European regions, by gender, mid-to-late 2000s.
159x207mm (300 x 300 DPI)

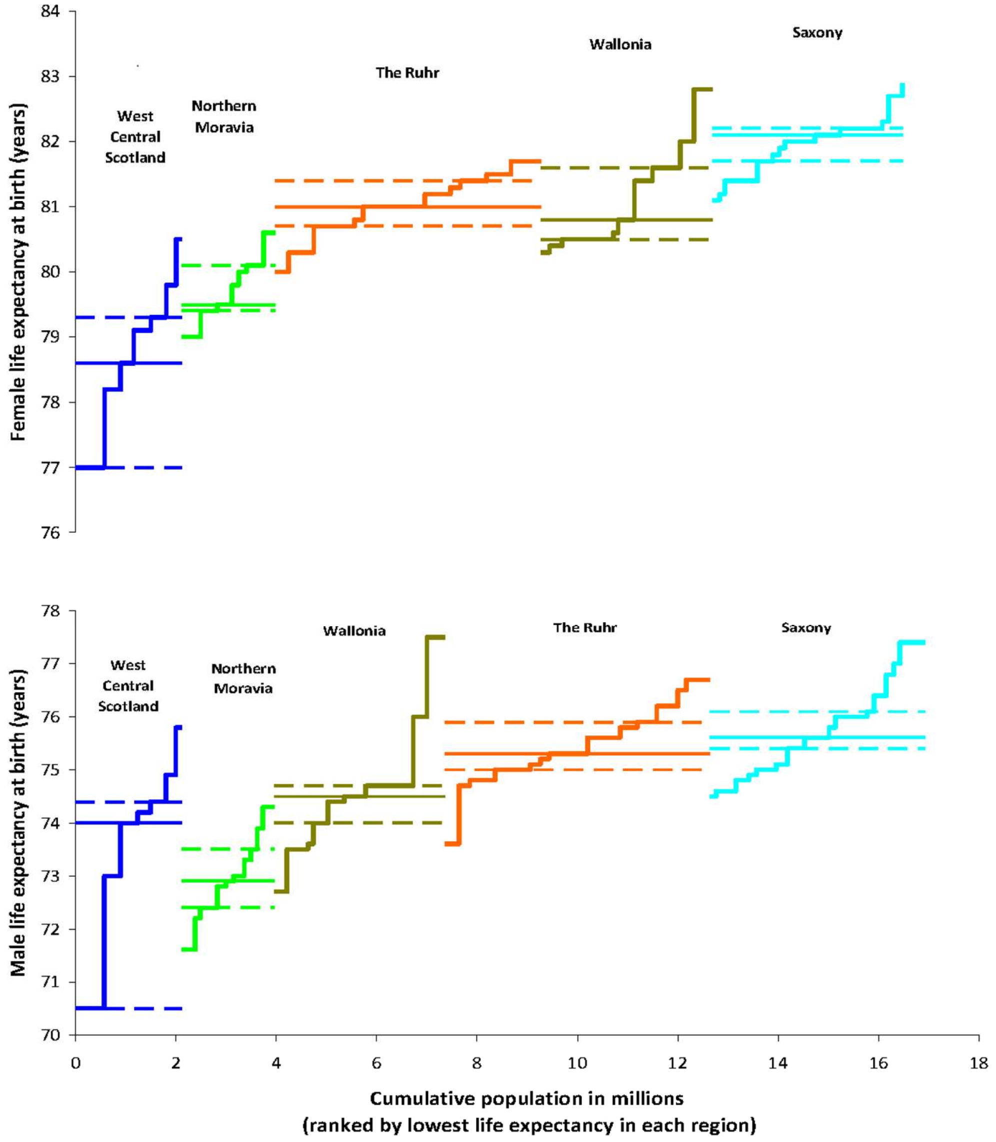

Figure 2: Inter-quartile range of life expectancy for large districts within five post-industrial European regions, by gender, mid-to-late 2000s.
159x185mm (300 x 300 DPI)

Web-only Appendix: Table 2: Sources for the life expectancy data

Region name	Downloaded or calculated?	Original source
West Central Scotland	Published data	National Records for Scotland (NRS), formerly General Register Office for Scotland.
Northern Ireland	Directly calculated by GCPH/NHS Health Scotland	Northern Ireland Statistics and Research Agency.
Merseyside	Published data	Office for National Statistics and Public Health Intelligence Team, Information & Intelligence Services, Liverpool Primary Care Trust - Liverpool Neighbourhood Management Areas Health Profiles 2010.
Swansea & South Wales Coalfields	Directly calculated by authors for Central Valleys; Published data for others	Office for National Statistics; Office for National Statistics (Vital Statistics).
Nord-Pas-de-Calais	Directly calculated by GCPH/NHS Health Scotland	INSEE and Centre d'épidémiologie sur les causes médicales de décès (CepiDc) – Data provided by Observatoire Régional de la Santé (ORS), NPdC.
Wallonia	Directly calculated by GCPH/NHS Health Scotland	SPMA (https://www.wiv-isp.be/epidemio/spma)<br>Public Health and Surveillance<br>Scientific Institute of Public Health, Brussels, Belgium.<br>https://www.wiv-isp.be/epidemio/spma/
The Ruhr	Published data	NRW Institute of Health and Work (LIGA.NRW).<br>http://www.lzg.gc.nrw.de<br>© Landesamt für Datenverarbeitung und Statistik NRW<br>Statistik der Sterbefälle<br>lögd: Sterbetafeln, Eigene Berechnung.
Saxony	Directly calculated by GCPH/NHS Health Scotland	Data provided by Statistisches Landesamt des Freistaates Sachsen.<br>© Statistisches Landesamt des Freistaates Sachsen, Kamenz, 2007.
Northern Moravia	Published data	Czech Statistical Office.<br>http://www.czso.cz/eng/redakce.nsf/i/life_tables<br>© Czech Statistical Office, 2012.
Silesia	Directly calculated by GCPH/NHS Health Scotland	Central Statistical Office of Poland – Local Data Bank.<br>http://www.stat.gov.pl/bdlen/app/strona.html?p_name=indeks<br>© 1995-2012 CSO.

For peer review only

STROBE Statement—checklist of items that should be included in reports of observational studies

	Item No	Recommendation
**Title and abstract**	1	(*a*) Indicate the study's design with a commonly used term in the title or the abstract
		(*b*) Provide in the abstract an informative and balanced summary of what was done and what was found
**Introduction**		
Background/rationale	2	Explain the scientific background and rationale for the investigation being reported
Objectives	3	State specific objectives, including any prespecified hypotheses
**Methods**		
Study design	4	Present key elements of study design early in the paper
Setting	5	Describe the setting, locations, and relevant dates, including periods of recruitment, exposure, follow-up, and data collection
Participants	6	(*a*) *Cohort study*—Give the eligibility criteria, and the sources and methods of selection of participants. Describe methods of follow-up<br>*Case-control study*—Give the eligibility criteria, and the sources and methods of case ascertainment and control selection. Give the rationale for the choice of cases and controls<br>*Cross-sectional study*—Give the eligibility criteria, and the sources and methods of selection of participants
		(*b*) *Cohort study*—For matched studies, give matching criteria and number of exposed and unexposed<br>*Case-control study*—For matched studies, give matching criteria and the number of controls per case
Variables	7	Clearly define all outcomes, exposures, predictors, potential confounders, and effect modifiers. Give diagnostic criteria, if applicable
Data sources/ measurement	8*	For each variable of interest, give sources of data and details of methods of assessment (measurement). Describe comparability of assessment methods if there is more than one group
Bias	9	Describe any efforts to address potential sources of bias
Study size	10	Explain how the study size was arrived at
Quantitative variables	11	Explain how quantitative variables were handled in the analyses. If applicable, describe which groupings were chosen and why
Statistical methods	12	(*a*) Describe all statistical methods, including those used to control for confounding
		(*b*) Describe any methods used to examine subgroups and interactions
		(*c*) Explain how missing data were addressed
		(*d*) *Cohort study*—If applicable, explain how loss to follow-up was addressed<br>*Case-control study*—If applicable, explain how matching of cases and controls was addressed<br>*Cross-sectional study*—If applicable, describe analytical methods taking account of sampling strategy
		(*e*) Describe any sensitivity analyses

Continued on next page

For peer review only - http://bmjopen.bmj.com/site/about/guidelines.xhtml

**Results**		
Participants	13*	(a) Report numbers of individuals at each stage of study—eg numbers potentially eligible, examined for eligibility, confirmed eligible, included in the study, completing follow-up, and analysed
		(b) Give reasons for non-participation at each stage
		(c) Consider use of a flow diagram
Descriptive data	14*	(a) Give characteristics of study participants (eg demographic, clinical, social) and information on exposures and potential confounders
		(b) Indicate number of participants with missing data for each variable of interest
		(c) *Cohort study*—Summarise follow-up time (eg, average and total amount)
Outcome data	15*	*Cohort study*—Report numbers of outcome events or summary measures over time
		Case-control study—Report numbers in each exposure category, or summary measures of exposure
		Cross-sectional study—Report numbers of outcome events or summary measures
Main results	16	(*a*) Give unadjusted estimates and, if applicable, confounder-adjusted estimates and their precision (eg, 95% confidence interval). Make clear which confounders were adjusted for and why they were included
		(*b*) Report category boundaries when continuous variables were categorized
		(*c*) If relevant, consider translating estimates of relative risk into absolute risk for a meaningful time period
Other analyses	17	Report other analyses done—eg analyses of subgroups and interactions, and sensitivity analyses
**Discussion**		
Key results	18	Summarise key results with reference to study objectives
Limitations	19	Discuss limitations of the study, taking into account sources of potential bias or imprecision. Discuss both direction and magnitude of any potential bias
Interpretation	20	Give a cautious overall interpretation of results considering objectives, limitations, multiplicity of analyses, results from similar studies, and other relevant evidence
Generalisability	21	Discuss the generalisability (external validity) of the study results
**Other information**		
Funding	22	Give the source of funding and the role of the funders for the present study and, if applicable, for the original study on which the present article is based

*Give information separately for cases and controls in case-control studies and, if applicable, for exposed and unexposed groups in cohort and cross-sectional studies.

Note: An Explanation and Elaboration article discusses each checklist item and gives methodological background and published examples of transparent reporting. The STROBE checklist is best used in conjunction with this article (freely available on the Web sites of PLoS Medicine at http://www.plosmedicine.org/, Annals of Internal Medicine at http://www.annals.org/, and Epidemiology at http://www.epidem.com/). Information on the STROBE Initiative is available at www.strobe-statement.org.

For peer review only
